# Inhibition of RACK1‐Mediated NLRP3 Oligomerization (Active Conformation) Ameliorates Acute Respiratory Distress Syndrome

**DOI:** 10.1002/advs.202411355

**Published:** 2025-05-11

**Authors:** Jian Cui, Meng Yang, Chengli Yu, Haidong Zhang, Yuan Gong, Yang Hu, Yue Wang, Qingxin Yuan, An Pan, Jiepin Li, Yaowen Hu, Zecheng Jin, Xuemei Peng, Anyuan Wu, Junwei Wang, Qian Wang, Yinan Zhang, Lihong Hu

**Affiliations:** ^1^ Jiangsu Key Laboratory for Functional Substance of Chinese Medicine Stake Key Laboratory Cultivation Base for TCM Quality and Efficacy School of Pharmacy Nanjing University of Chinese Medicine Nanjing 210023 China; ^2^ Department of Respiratory Medicine Affiliated Hospital of Nanjing University of Chinese Medicine Jiangsu Province Hospital of Chinese Medicine Nanjing Jiangsu 210029 China; ^3^ China Joint Graduate School of Traditional Chinese Medicine Suzhou Jiangsu 215105 China

**Keywords:** ARDS, bigelovin, NLRP3 inflammasome, RACK1

## Abstract

Aberrant activation of the NACHT, LRR, and PYD domain‐containing protein 3 (NLRP3) inflammasome contributes to the pathogenesis of fatal and perplexing pulmonary diseases. Although pharmacological inhibition of the NLRP3 inflammasome brings potent therapeutic effects in clinical trials and preclinical models, the molecular chaperones and transition governing its transformation from an auto‐suppressed state to an active oligomer remain controversial. Here, this work shows that sesquiterpene bigelovin inhibited NLRP3 inflammasome activation and downstream pro‐inflammatory cytokines release via canonical, noncanonical, and alternative pathways at nanomolar ranges. Chemoproteomic target identification discloses that bigelovin covalently bound to Cys168 of RACK1, disrupting the interaction between RACK1 and NLRP3 monomer and thereby suppressing NLRP3 inflammasome oligomerization in vitro and in vivo. Bigelovin treatment significantly alleviates the severity of NLRP3‐related pulmonary disorders in murine models, such as LPS‐induced ARDS and silicosis. These results consolidated the intricate role of RACK1 in transiting the NLRP3 state and provided a new anti‐inflammatory lead and therapy for NLRP3‐driven diseases.

## Graphical abstract

Schematic diagram showing the potential mechanism of bigelovin on the activation of NLRP3 inflammasome Bigelovin may inhibit activated protein C kinase 1 (RACK1) by directly binding with cys168 of RACK1. Bigelovin thus prevents oligomerization of NLRP3 (NLRP3 active conformation) and subsequent assembly of NLRP3 inflammasome, blocking the activation of canonical, non‐canonical, and alternative NLRP3 inflammasome.

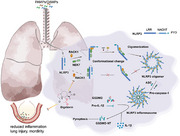

## 1 Introduction

Acute respiratory distress syndrome (ARDS), characterized by acute hypoxemia, diffuse lung inflammation, and edema, is the primary cause of respiratory failure with more than 40% mortality.^[^ ^1^, ^2^ ^]^ Few therapeutic modalities are presented to alleviate this deadly condition. The disorder is associated with excessive alveolar capillary permeability and diffuse damage. Most ARDS cases initiate in patients exposed to pathogen‐associated molecular patterns (such as LPS or RNA) during bacterial and viral types of pneumonia.^[^ ^3^ ^]^ There is growing evidence of the involvement of innate immunity during ARDS pathogenesis, in which alveolar macrophages detect the presence of infections or injuries and trigger pyroptosis characterized by rapid cytolysis and the release of proinflammatory cytokines.^[^ ^4^, ^5^ ^]^ Based on our and other groups’ study, an overproduction of IL‐1β caused by macrophage pyroptosis in the development of ARDS is essential for activating the host's immune system.^[^ ^6^, ^7^ ^]^ Therefore, targeting the production of IL‐1β might be beneficial to ARDS.

As the core signaling to drive IL‐1β activation and release, inflammasomes assembled by multiple proteins play a crucial role in inflammatory response to infection and tissue damage. Inflammasome complexes generally consist of pattern recognition receptors (PRRs), the adaptor protein apoptosis‐associated speck‐like protein containing a CARD (ASC), and the inflammatory proteases caspase‐1.^[^ ^8^ ^]^ The most extensively studied pattern recognition receptor (PRR)‐NLRP3 inflammasome, is an intracellular sensor for infectious and sterile stress signals, which deeply takes part in triggering inflammatory responses and mediating pyroptosis.^[^ ^9^ ^]^ Its activation primarily depends on the priming step, typically via TLR‐induced NF‐κB‐dependent signaling pathways, which upregulates the expression of *Nlrp3* and facilitates subsequent inflammasome assembly. Additionally, direct LPS treatment could also prime NLRP3 in a non‐transcriptional way. In the activation step, NLRP3 is activated by diverse stimuli ranging from bacterial toxins to particulates that often converge to ionic changes such as K^+^ efflux or Ca^2+^ influx.^[^ ^10^, ^11^ ^]^ A number of NLRP3‐binding proteins have been found to promote NLRP3 inflammasome translocation and assembly.^[^ ^12^ ^]^ For example, SCAP and SREBP2 interact with the NACHT domain and help NLRP3 transfer to the Golgi apparatus^[^ ^13^ ^]^; post‐translational phosphorylation at S293 of NLRP3 by protein kinase D allows the release from the Golgi and the formation of cytosolic inflammasome^[^ ^14^ ^]^; deficiency of another mediator HDAC6 traps NLRP3 as small speckles at the trans‐Golgi network and stops its transportation to the centrosome where assembly takes place.^[^ ^15^ ^]^ On the other hand, NEK7 is the most‐studied mediator to trigger the final formation of NLRP3 inflammasome machinery,^[^ ^16^ ^]^ in which structural analysis of NLRP3‐NEK7 complex shows that NEK7 bridges adjacent subunits in the process of NLRP3 oligomerization.^[^ ^17^ ^]^ Next, the receptor for activated protein C kinase (RACK1) interacts with NLRP3 and NEK7, leading NLRP3 into an “active” conformation in response to stimuli and subsequent inflammasome assembly.^[^ ^18^ ^]^

Since NLRP3 inflammasome and its regulatory role have emerged as a promising therapeutic target, the development of direct NLRP3 inhibitors is vigorously progressing in the preclinical and clinical stages. However, whether these partner proteins are related to the inflammasome structure and post‐translational modification remains unclear. Our group previously demonstrated that EZH2 promotes the expression of autophagy‐related protein 5, which constitutes a novel NLRP3 degradation pathway. Furthermore, we found that the flavonoid lonicerin acts as an EZH2 inhibitor, thereby alleviating excessive inflammation in the ulcerative colitis model.^[^ ^19^ ^]^ During our search for natural products targeting the NLRP3 inflammasome and its mediators, we identified sesquiterpene bigelovin as a covalent binder that blocks RACK1‐mediated NLRP3 oligomerization, using an isoTOP‐ABPP chemoproteomic approach.^[^ ^20^ ^]^ Here, we report the discovery of a RACK1 inhibitor that exerts NLRP3 inflammasome antagonistic activity in vitro and in vivo. Mechanistically, sesquiterpene lactone bigelovin covalently binds to a cysteine residue of RACK1 and blocks NLRP3 oligomerization (active conformation), inhibiting the assembly of NLRP3 inflammasome, caspase‐1 activation, and IL‐1β production, which ultimately reduces ARDS severity.

## 2 Results

### 2.1 Sesquiterpene Lactone Contained in *Inula helianthus‐aquatica* Reduces LPS‐Induced ARDS in Mice

During the COVID‐19 pandemic, ARDS emerged as the predominant syndrome and the leading cause of death among severe COVID‐19 patients.^[^ ^21^ ^]^ Both clinical and preclinical studies have delineated the effectiveness of traditional Chinese medicine (TCM) in treating COVID‐19 and its associated complications.^[^ ^22^ ^]^ The TCM formulae containing the flowers of *Inula helianthus‐aquatica* have been traditionally used in the prescriptions of lung diseases, such as asthma and chronic bronchitis.^[^ ^23^ ^]^ The anti‐inflammatory properties of the defining components in Inula helianthus‐aquatica remain unproven. To pinpoint the active components in the plant, we initially employed an activity‐guided isolation approach to refine the fractions derived from crude extraction. Mice received two doses of the fractions on consecutive days before a single intraperitoneal injection of 7.5 mg kg^−1^ LPS (Figure S1B, Supporting Information). We evaluated pulmonary inflammation by measuring the mRNA levels of pro‐inflammatory cytokines in the lungs at 12 and 24 h after LPS delivery. The results showed that the fraction containing sesquiterpene lactones bigelovin, ergolide, and 8‐epi‐helenalin (Figure S1A, Supporting Information), significantly decreased the expression of IL‐1β in the lungs at both 1 mg kg^−1^ and 0.1 mg kg^−1^ doses, while showing minimal effects on IL‐6 and TNF‐α levels. (Figure S1C–G, Supporting Information). To confirm our results, we assessed whether the same fraction could attenuate the LPS‐induced release of pro‐inflammatory cytokines (Figure S1H, Supporting Information). Consistent with the preventative remedy, post‐LPS challenge administration also markedly reduced lung inflammation at 1 mg kg^−1^ compared to dexamethasone at 5 mg kg^−1^ (Figure S1I–M, Supporting Information). These findings suggest that sesquiterpene lactones present in the extract of *Inula helianthus‐aquatica* may mitigate the severity of ARDS and reduce pulmonary inflammation.

### 2.2 Aberrant Activation of Macrophage‐NLRP3 Inflammasome Activation is Associated with ARDS

**Figure 1:**
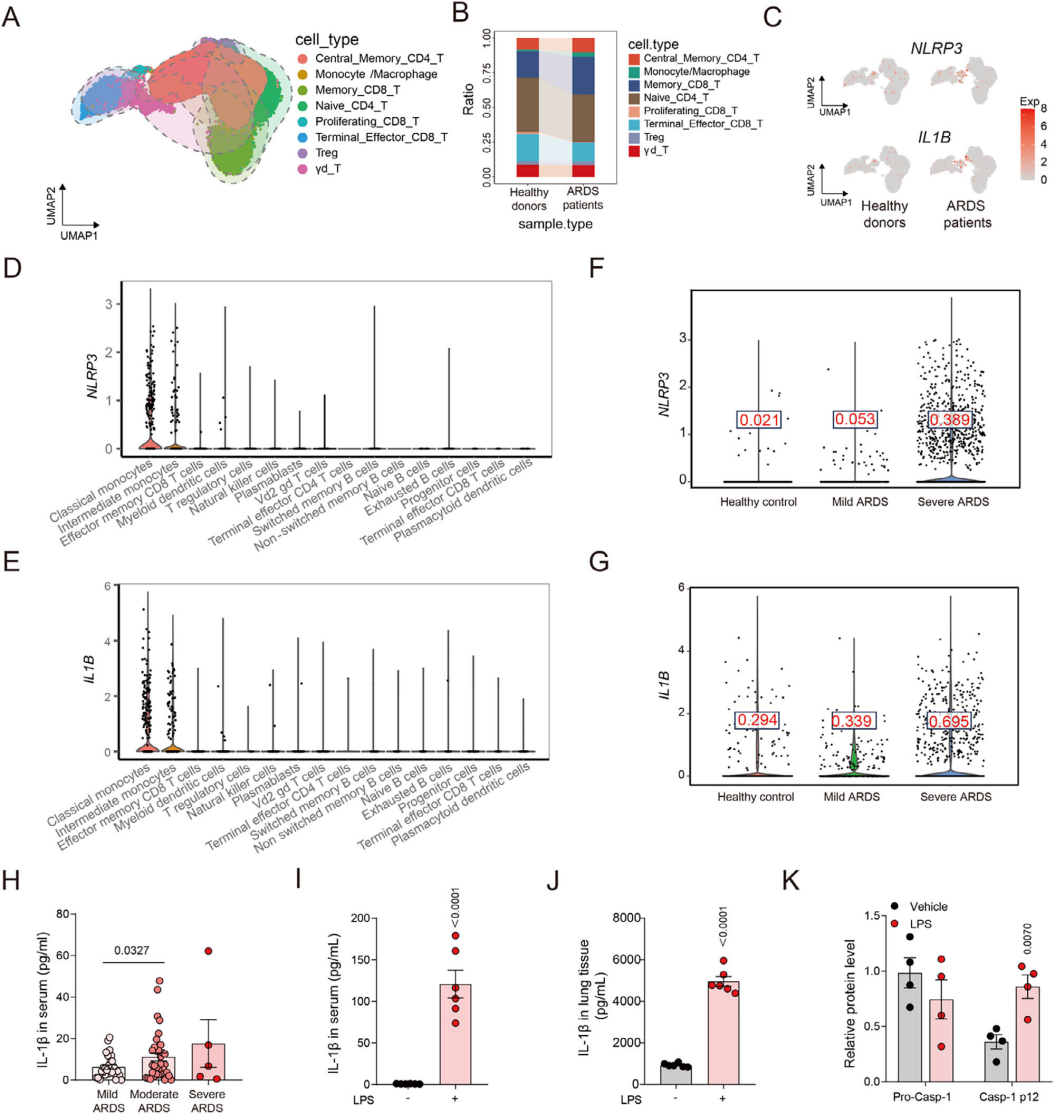
Aberrant activation of NLRP3 inflammasome in macrophages during ARDS pathogenesis. A) Uniform Manifold Approximation and Projection (UMAP) plot showing immune cell from GSE175450. B) The proportion of each cell type in PBMCs from healthy donors and ARDS patients in single‐cell data (GSE175450). C) UMAP plots show the expression of NLRP3 and IL‐1B in PBMCs from healthy donors and ARDS patients (GSE175450). The redder the color, the higher the expression levels are. (D and E) Expression of *NLRP3* D) and *IL1B* E) in BALF in each immune cell type among severe ARDS (GSE145926). F,G) Expression of *NLRP3* (F) and *IL1B* (G) in BALF macrophages cells among healthy controls (*n* = 3) and patients with mild (*n* = 3) and severe ARDS (*n* = 6) patients (GSE145926). The red numbers in the box represent the mean gene expression level. H) Concentrations of IL‐1β in serum from patients with direct acute respiratory distress syndrome (ARDS). Serum of patients with mild (*n* = 35) or indirect ARDS (*n* = 28) and severe ARDS (*n* = 5) was obtained and analyzed for IL‐1β. I,J) C57BL/6J mice were challenged with 7.5 mg kg^−1^ LPS for 12 h. The levels of IL‐1β in serum (I) or lung tissues (J) from mice were measured by ELISA (*n* = 6). K) Western blotting analysis of caspase‐1 (p12) from above mice (*n* = 4). Data were presented as mean ± SEM and statistical significance was assessed by two‐tailed unpaired *t* test.

The NLRP3 inflammasome is pivotal in the innate immune system's response to escalating inflammatory stimuli. To figure out the expression profile of the NLRP3 inflammasome in ARDS, we commenced by analyzing single‐cell RNA sequencing data from peripheral blood mononuclear cells (PBMCs) of thirteen ARDS patients and six healthy donors, as reported by *Sawitzki* et al. ^[^ ^24^ ^]^ The Human Protein Atlas single‐cell database showed that NLRP3 was primarily expressed in myeloid cells (Figure S2A, Supporting Information). Quantitative analysis demonstrated a significant increase in monocyte/macrophage density in ARDS patients compared to healthy controls (**Figure** **1**A,B). Consistent with this finding, peripheral blood samples from ARDS patients exhibited markedly elevated levels of NLRP3 and IL‐1β relative to healthy donors (Figure 1C; Figure S2B, Supporting Information). Given that ARDS is characterized by lung inflammation arising from activation of the innate immune system, we next examined the expression patterns of NLRP3 and IL‐1β through scRNA‐seq analysis of bronchoalveolar lavage fluid (BALF) cells obtained from individuals with severe ARDS. Our findings revealed that the expression of NLRP3 and IL‐1β genes is predominantly observed in classical and intermediate monocytes in BALF (Figure 1D,E). Further analysis of specific gene expression in BALF was conducted. Notably, the single‐cell RNA sequencing of BALF also disclosed an elevated expression of NLRP3 and IL‐1β in monocytes of severe ARDS compared to those with moderate ARDS and healthy controls (Figure 1F,G). To further validate these observations, we directly evaluated NLRP3 inflammasome activation in both ARDS patients and an LPS‐induced murine ARDS model. The data indicated a direct correlation between IL‐1β serum levels and the severity of the condition (Figure 1H; Table 1.j Patient Characteristics with Direct ARDS). Specifically, the LPS‐induced ARDS murine model demonstrated a similar trend in IL‐1β secretion in serum and lung tissue (Figure 1I,J). Western blotting analyses of caspase‐1 signaling indicated pronounced cleavage and activation of pro‐caspase‐1 in the ARDS model (Figure 1K; Figure S2C, Supporting Information), validating the aberrant activation of NLRP3 inflammasome in ARDS.

### 2.3 Bigelovin Specifically Abrogates NLRP3 Inflammasome Activation In Vitro and In Vivo

**Figure 2:**
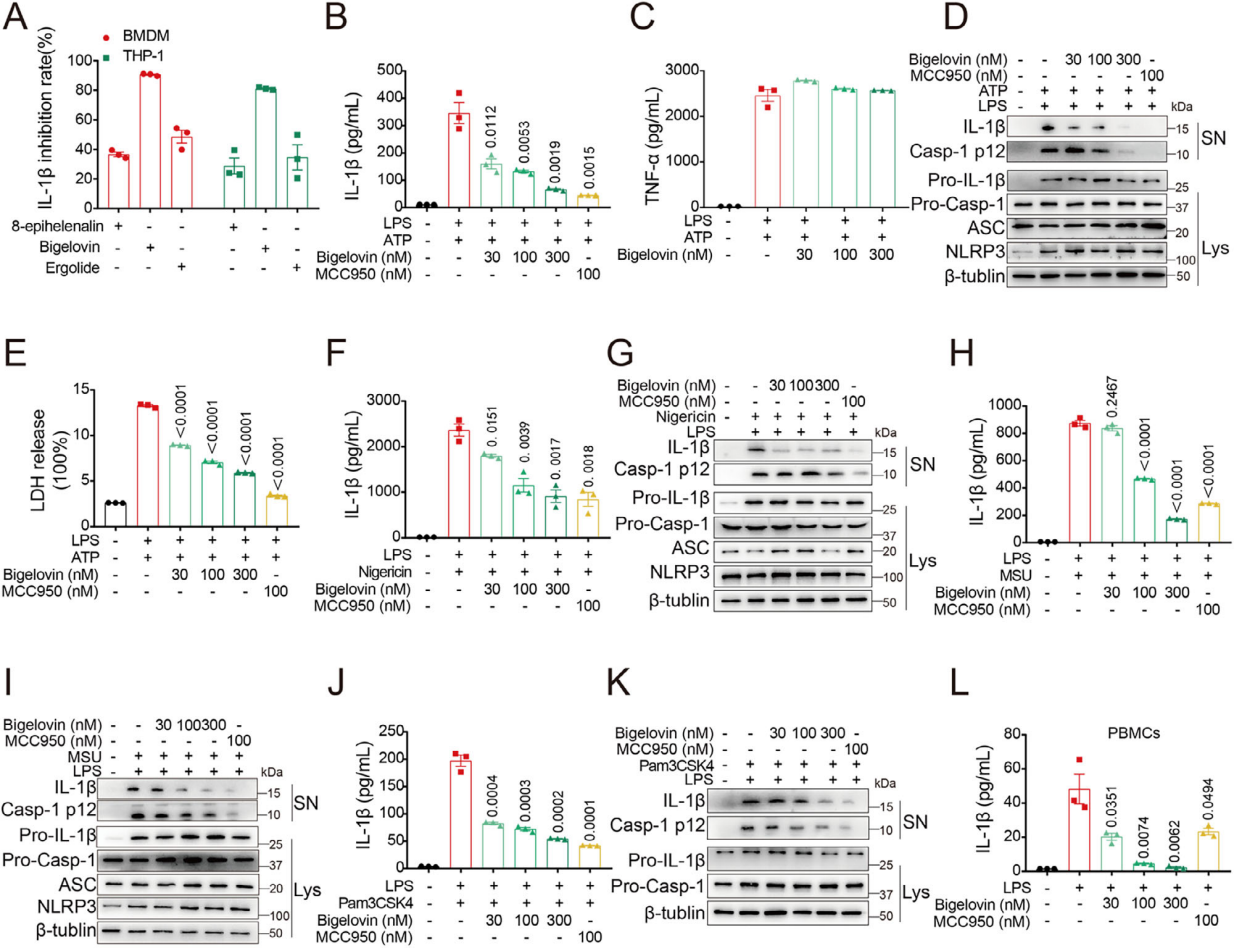
Bigelovin potently inhibits inflammasome activation. A) ELISA analysis of IL‐1β in culture supernatants of LPS‐primed BMDMs or THP‐1 treated with 8‐epihelenalin, bigelovin, or ergolide (1 µM) before stimulation with ATP (5 mM, 45 min). B–E) LPS‐primed BMDMs treated with bigelovin before stimulation with ATP (5 mM, 45 min). ELISA analysis of IL‐1β (B) or TNF‐α (C) in culture supernatants. D) Western blotting analysis of IL‐1β (p17) and caspase‐1 (p12) in culture supernatants (SN) and pro‐IL‐1β and pro‐caspase‐1 in lysates (Lys). E) Assay of LDH release in culture supernatants. F,G) LPS‐primed BMDMs treated with bigelovin before stimulation with Nigericin. (F) ELISA analysis of IL‐1β in culture supernatants. G) Western blotting analysis of IL‐1β (p17) and caspase‐1 (p12) in culture supernatants (SN) and pro‐IL‐1β and pro‐caspase‐1 in lysates (Lys). H,I) LPS‐primed BMDMs treated with bigelovin before stimulation with MSU. H) ELISA analysis of IL‐1β in culture supernatants. I) Western blotting analysis of IL‐1β (p17) and caspase‐1 (p12) in culture supernatants (SN) and pro‐IL‐1β and pro‐caspase‐1 in lysates (Lys). J,K) BMDMs are primed with Pam3CSK4 and treated with bigelovin before transfected with LPS. J) ELISA analysis of IL‐1β in culture supernatants. K) Western blotting analysis of IL‐1β (p17) and caspase‐1 (p12) in culture supernatants (SN) and pro‐IL‐1β and pro‐caspase‐1 in lysates (Lys). L) ELISA analysis of IL‐1β in culture supernatants of PBMCs treated with bigelovin and then stimulated with LPS. Data were presented as mean ± SEM and were representative of three independent experiments. Statistical significance was assessed by two‐tailed unpaired *t* test.

In the lung, NLRP3 was particularly expressed in macrophages (Figure S2D,E, Supporting Information). Subsequently, we explored the inhibitory effects of bigelovin, ergolide, and 8‐epi‐helenalin on IL‐1β production in mouse bone marrow‐derived macrophages (BMDMs) and human monocytic THP‐1‐derived macrophages at non‐toxic concentrations. The results showed that bigelovin exerted the most potent suppression of IL‐1β production at a concentration of 1 µM in these LPS/ATP‐stimulated cells, compared to the other sesquiterpene lactones (**Figure** **2A**; Figure S3A–F, Supporting Information). The IC50 values for bigelovin were determined at 46.0 nM and 396.8 nM in BMDMs and THP‐1 cells, respectively (Figure S3G,H, Supporting Information). Within the effective range, bigelovin did not affect levels of other pro‐inflammatory cytokines such as TNF‐α and IL‐6, suggesting its specific inhibition to the NLRP3 pathway (Figure 2B,C; Figure S3I–K, Supporting Information). In addition to IL‐1β maturation, caspase‐1 activation is also implicated in cell pyroptosis and subsequent release of lactate dehydrogenase (LDH) during the activation of NLRP3 inflammasome. Bigelovin dose‐dependently inhibited caspase‐1 cleavage and LDH release (Figure 2D,E; Figure S3L,M, Supporting Information). This inhibitory effects on IL‐1β production and caspase‐1 activation were consistent across BMDMs and human macrophages when challenged with various NLRP3 inflammasome activators, including nigericin and monosodium urate crystals (MSU) (Figure 2F–I; Figure S3N–S, Supporting Information). To further elucidate the inhibitory mechanism of bigelovin, we examined its effect on noncanonical NLRP3 inflammasome activation induced by intracellular LPS. In line with our previous observations, bigelovin significantly reduced IL‐1β production and caspase‐1 activation in Pam3CSK4‐primed BMDMs challenged with LPS (Figure 2J,K; Figure S3T,U, Supporting Information). In monocytes, LPS alone can trigger NLRP3 inflammasome signaling. We also observed that bigelovin decreased the secretion of IL‐1β in PBMCs induced by LPS in a dose‐dependent manner (Figure 2L; Figure S3V, Supporting Information), illustrating its broad‐spectrum inhibitory effects on the NLRP3 inflammasome across diverse cell types.

**Figure 3:**
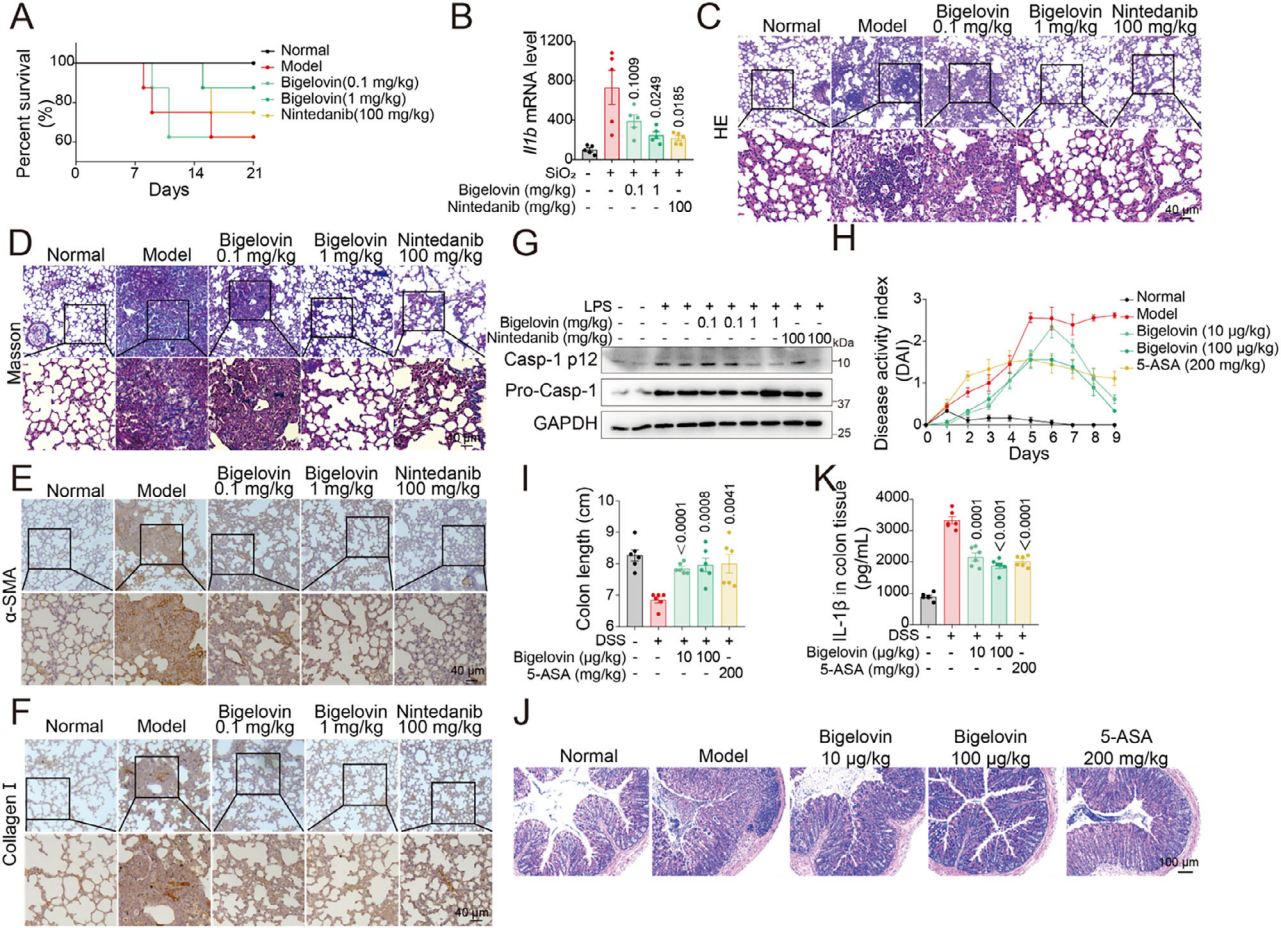
Bigelovin inhibits NLRP3 inflammasome activation in vivo. A) Mice were evaluated for changes in survival treated with bigelovin (0.1 and 1 mg kg^−1^) or nintedanib (100 mg kg^−1^) (*n* = 8 per group). B) qRT‐PCR analysis of *Il1b* mRNA expression in lung tissues of mice (*n* = 5). C,D) Representative H&E‐stained sections (C) or Masson staining (D) of lung tissues are shown (scale bar = 40 µm). E,F) Lung sections from above mice were stained with α‐SMA (E) and Collagen I (F) antibodies, (scale bar = 40 µm). G) Western blotting analysis of caspase‐1 (p12) in the lung from above mice. H–K) Mice were treated with 2.5% DSS dissolved in the drinking water for 7 days and were then provided normal drinking water for 2 days. Bigelovin (10, 100 µg kg^−1^) or vehicle were intraperitoneal injection daily (*n* = 6). The disease activity index (DAI) (H), and colon length (I) were measured. J) Sections of paraffin‐embedded colon tissues were stained with H&E (Scale bar = 100 µm). K) Colon IL‐1β levels were assessed by ELISA. Data were presented as mean ± SEM and statistical significance was assessed by two‐tailed unpaired *t* test.

To further validate the role of bigelovin in inflammasome activation in *vivo*, we employed the murine silicosis model, an NLRP3‐dominant disease model^[^ ^25^ ^]^ (Figure S4A, Supporting Information). Our result revealed that administering bigelovin at a low dosage (1 mg kg^−1^) dramatically enhanced survival rates from 62.5% to 87.5%. Notably, the positive control nintedanib showed reduced efficacy even at a much higher dosage (**Figure** **3A**). In the measurement of pro‐inflammatory cytokines, it was evident that IL‐1β levels were markedly reduced in the bigelovin‐treated group (Figure 3B). Histopathological analysis of alveolar tissues from the model and treatment groups revealed less lung tissue damage, characterized by reduced infiltration of septal mononuclear cells, lymphocytes, alveolar macrophages, neutrophils, and diminished alveolar edema (Figure 3C,D). Furthermore, bigelovin effectively curtailed collagen accumulation, as indicated by the staining of α‐SMA and collagen I (Figure 3E,F). Immunoblotting analysis for caspase‐1 corroborated these observations, illustrating that bigelovin significantly impeded the cleavage of caspase‐1, and activation of the NLRP3 inflammasome in a dose‐dependent manner (Figure 3G; Figure S4B, Supporting Information). Besides, we also determined the NLRP3 inflammasome inhibitory role in dextran sulfate sodium (DSS)‐induced colitis. In our results, bigelovin markedly inhibited the weight loss and disease activity index (DAI) of mice induced by DSS in a dose‐dependent manner (Figure 3H; Figure S4C, Supporting Information). In addition, colon shortening was also inhibited by bigelovin (Figure 3I). Histological analysis and qRT‐PCR analysis also revealed that bigelovin treatment reduced the destruction of the intestinal epithelium and infiltration of immune cells (Figure 3J; Figure S4D–F, Supporting Information). Additionally, the level of IL‐1β in the colon was also decreased by bigelovin, suggesting the inhibitory effect of bigelovin on the NLRP3 inflammasome in *vivo* (Figure 3K). Collectively, these findings exhibited that bigelovin could block the activation of NLRP3 inflammasome in vivo and mitigate the severity of silicotic and colitis mice.

### 2.4 Bigelovin Reduces LPS‐Induced Acute Respiratory Distress Syndrome

**Figure 4:**
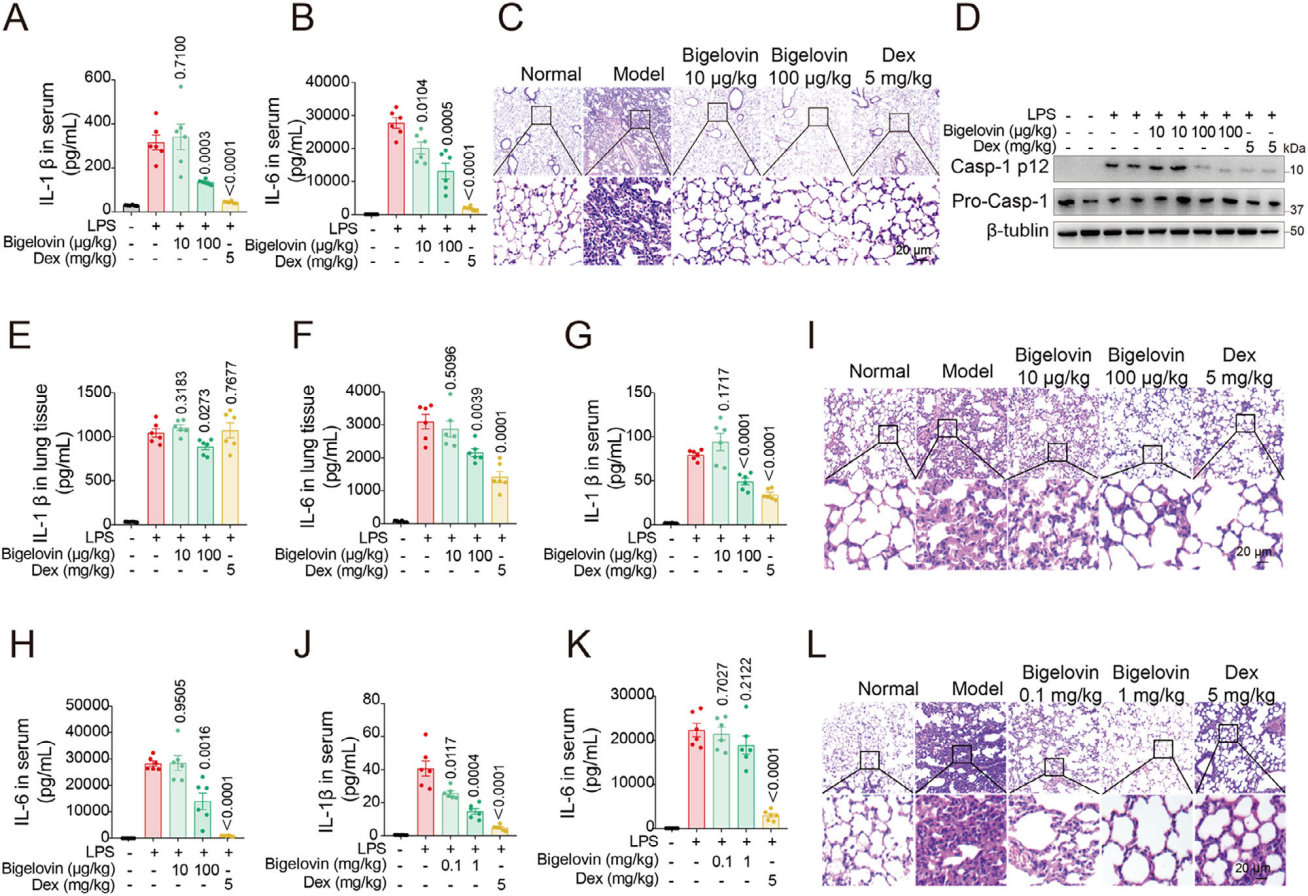
Bigelovin improves LPS‐induced ARDS. A,B) Mice were prophylactic intraperitoneal injection of bigelovin (10 µg kg^−1^, 100 µg kg^−1^) or dexamethasone (5 mg kg^−1^) then challenged with 7.5 mg kg^−1^ LPS for 12 h. IL‐1β (A) and IL‐6 (B) levels of serum from above mice were measured by ELISA (*n* = 6). C) H&E staining of lung tissues from mice that were untreated or pretreated with bigelovin and then challenged with 7.5 mg kg^−1^ LPS for 24 h prior tissue collection (scale bar = 20 µm). D) Mice were prophylactic intraperitoneal injection of bigelovin (10 µg kg^−1^, 100 µg kg^−1^) or dexamethasone (5 mg kg^−1^) then challenged with 7.5 mg kg^−1^ LPS for 12 h. Western blotting analysis of caspase‐1 (p12) from above mice (*n* = 5). E–I) Mice were treated with 7.5 mg kg^−1^ LPS and then injected intraperitoneally bigelovin (10 µg kg^−1^, 100 µg kg^−1^) or dexamethasone (5 mg kg^−1^) for 24 h. IL‐1β (E) and IL‐6 (F) levels of lung tissues from above mice were measured by ELISA (*n* = 6). IL‐1β (G) and IL‐6 (H) levels of serum were measured by ELISA (*n* = 6). I) H&E staining of lung tissues from above mice that were treated with 7.5 mg kg^−1^ LPS (scale bar = 20 µm). J,K) Mice were prophylactic intragastric administration of bigelovin (0.1 mg kg^−1^, 1 mg kg^−1^) or dexamethasone (5 mg kg^−1^) and then challenged with 7.5 mg kg^−1^ LPS for 12 h. IL‐1β (J) and IL‐6 (K) levels of serum from above mice were measured by ELISA (*n* = 6). L) H&E staining of lung tissues from mice that were prophylactic intragastric administration of bigelovin and then challenged with 7.5 mg kg^−1^ LPS for 24 h (scale bar = 20 µm). Data were presented as mean ± SEM and statistical significance was assessed by two‐tailed unpaired *t* test.

Considering the pivotal role of the NLRP3 inflammasome in ARDS, we next focused on whether bigelovin could prevent or mitigate ARDS in mice. Prior studies have shown that LPS administration could trigger the production of pro‐inflammatory cytokines such as IL‐1β, IL‐6, and TNF‐α, and the recruitment of inflammatory cells like macrophages and neutrophils into the lungs, which further leads to damage to the pulmonary vascular endothelium, interstitial edema, recruitment of innate immune cells into the septa and alveolar space, resulting in the thickening of alveolar walls and secondary collagen deposition.^[^ ^26^ ^]^ In our study, mice received either vehicle or bigelovin at two dose levels (0.01 and 0.1 mg kg^−1^) via intraperitoneal injection once daily for three consecutive days, with the final administration given 30 min prior to the LPS challenge. Our observations indicated the bigelovin‐treated groups showed a dose‐dependent reduction in septal thickening and infiltration of monocytic cells into the bronchioles, vascular bed, and lung parenchyma. Notably, a dosage of as low as 0.1 mg kg^−1^ was sufficient to confer robust protection (**Figure** **4**A–C; Figure S5A–G, Supporting Information). Furthermore, this treatment approach significantly decreased the levels of caspase‐1 (Figure 4D; Figure S5L, Supporting Information).

In our continued investigation of the therapeutic potential of bigelovin, we administered the same dosages (0.1 and 1 mg kg^−1^) to the LPS‐induced ARDS mice at 30 min and 24 h post‐LPS challenge. It was demonstrated that both respiratory symptoms and pro‐inflammatory cytokine levels were alleviated, matching the efficacy of prophylactic treatment (Figure 4E–I; Figure S5H,I, Supporting Information). To account for the differences in the administrative routes, we further examined the preventive effect of bigelovin orally. While a dose of 0.1 mg kg^−1^ yielded a modest protective effect, increasing the dose to 1 mg kg^−1^ significantly enhanced protection compared to the injection method (Figure 4J–L; Figure S5J,K, Supporting Information). These data together demonstrated that bigelovin treatment can prevent and mitigate LPS‐induced ARDS in mice. Further toxicity assessment of bigelovin (up to 10 mg kg^−1^, i.g., for 30 days) revealed no adverse effects in mice (Figure S6A–F, Supporting Information).

### 2.5 Bigelovin Prevents the Assembly of NLRP3 Inflammasome Induced by the Oligomerization of NLRP3

**Figure 5:**
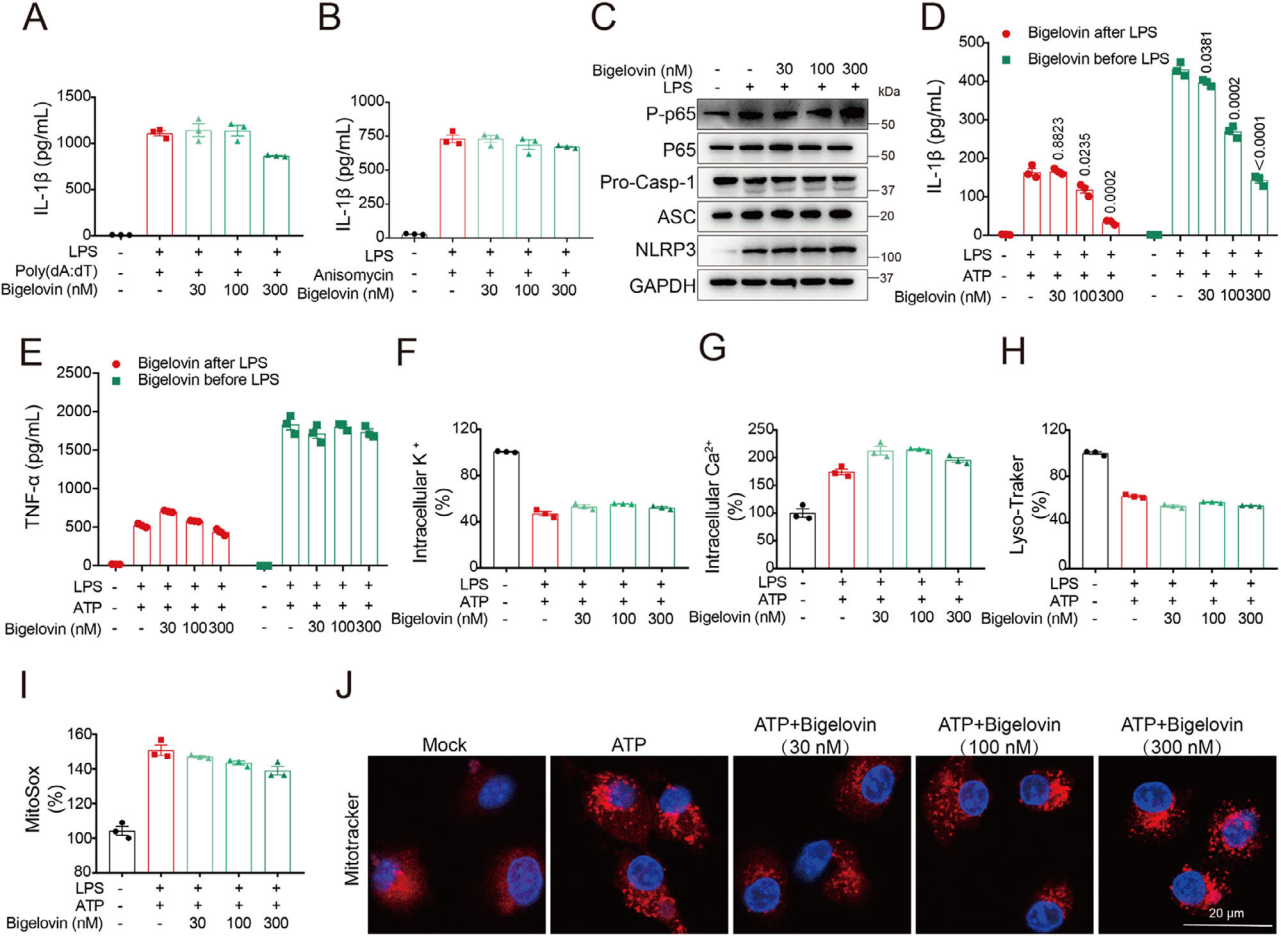
Bigelovin specifically inhibits NLRP3 inflammasome activation and does not affect the upstream pathways of the NLRP3 inflammasome. A,B) ELISA analysis of IL‐1β from LPS‐primed BMDMs treated with bigelovin before stimulation with poly(dA:dT) (A) or anisomycin (B). C) Western blotting analysis of p‐p65, p65, pro‐caspase‐1, ASC, NLRP3 and GAPDH in LPS‐primed BMDMs pretreated with bigelovin. (D and E) ELISA analysis of IL‐1β (D) or TNF‐α (E) in culture supernatants of BMDMs treated with bigelovin before or after LPS challenged and stimulated with ATP. F–I) LPS‐primed BMDMs were treated with bigelovin and then stimulated with ATP. Flow cytometric analysis of K^+^ efflux (F), Ca^2+^ influx (G), Lysosome rupture (H) and mitochondrial reactive oxygen species (I). J) Confocal immunofluorescence images of LPS‐primed BMDMs treated with BMDMs and stimulated with ATP, followed by staining with Mitotracker and DAPI (scale bar = 20 µm). Data were presented as mean ± SEM and were representative of three independent experiments. Statistical significance was assessed by two‐tailed unpaired *t* test.

In the selective analysis, we assessed bigelovin's inhibitory effects on other inflammasomes, such as AIM2 and NLRP1. The results showed that bigelovin did not affect the activation of AIM2 inflammasome induced by poly(dA:dT) and NLRP1 inflammasome activated by anisomycin, suggesting that bigelovin specifically inhibited NLRP3 inflammasome activation (**Figure** **5**A,B). We then shifted our attention to the inhibitory mechanism of bigelovin on the NLRP3 inflammasome by detecting the priming and activation stage of the NLRP3 inflammasome complex. The results showed that bigelovin could not inhibit NF‐κB activation and LPS‐induced transcription of *Nlrp3*, *Il1b*, *Tnfa*, and *Il6* at valid concentrations (0.03–0.3 µM) in BMDMs before LPS treatment (Figure 5C; Figure S7A–F, Supporting Information). Additionally, bigelovin did not affect the expression of the components of the NLRP3 inflammasome machinery (Figure 5C; Figure S7B, Supporting Information). Bigelovin potently blocked IL‐1β production in BMDMs irrespective of LPS stimulation timing, but only marginally affected TNF‐α release (Figure 5D,E). Collectively, these results illustrated that bigelovin did not affect Toll‐like receptor signaling or NLRP3 priming at the doses.

**Figure 6:**
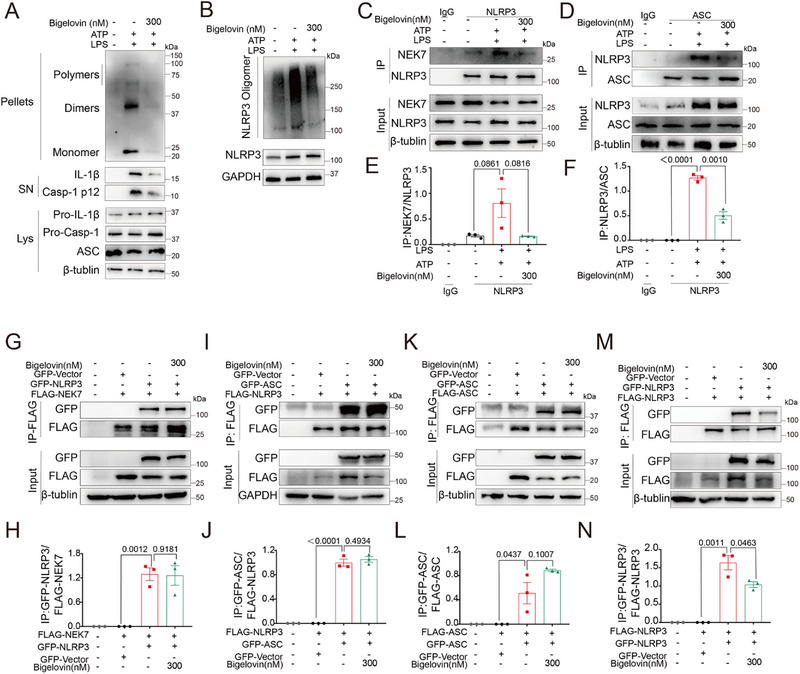
Bigelovin inhibits NLRP3 inflammasome assembly by blocking NLRP3‐NLRP3 interaction. A–F) LPS‐primed BMDMs were treated with 300 nM bigelovin and then stimulated with ATP. Immunoblot analysis of ASC oligomerization (A) and NLRP3 oligomerization (B). Immunoprecipitation analysis of the interaction between NLRP3 and NEK7 (C) or ASC (D). Quantitative analysis of the interaction between NLRP3 and NEK7 (E) or ASC (F). G–N) Immunoprecipitation analysis of the interaction of between NLRP3 and NEK7 (G), or ASC (I), the interaction of ASC and ASC (K) and the interaction of NLRP3 and NLRP3 (M) in HEK‐293T cells transfected with indicated plasmid and treated with bigelovin (300 nM) for 24 h. Quantitative analysis of the interaction of GFP‐NLRP3 and FLAG‐NEK7 (H) described in (G), the interaction of GFP‐ASC and FLAG‐NLRP3 (J) described in (I), the interaction of GFP‐ASC and FLAG‐ASC (L) described in (K) and the interaction of GFP‐NLRP3 and FLAG‐NLRP3 (N) described in (M). Data were presented as mean ± SEM and were representative of three independent experiments. Statistical significance was assessed by two‐tailed unpaired *t* test.

Since bigelovin was ineffective in the upstream events leading to NLRP3 inflammasome activation, such as K^+^ efflux and Ca^2+^ influx, the damage to lysosome and mitochondria (Figure 5F–J), we questioned whether it played a potential role in the assembly of NLRP3 inflammasome. Initial assessments of bigelovin's impact on the oligomerization of ASC and NLRP3 revealed a strong inhibition of this process at a concentration of 0.3 µM in ATP‐stimulated BMDMs (**Figure** **6**A,B). Furthermore, immunoprecipitation studies showed that bigelovin significantly blocked the endogenous interaction between NLRP3 and its partners NEK7/ASC (Figure 6C–F). To characterize its specific role within the NLRP3 inflammasome assembly, we profiled ATP‐dependent NLRP3 inflammasome assembly intermediates, including NLRP3‐NEK7 binding, NLRP3‐ASC recruitment, and ASC speck formation in HEK293T cells. Treatment with 0.3 µM bigelovin did not alter the exogenous interaction between NLRP3 and NEK7/ASC, nor the self‐interaction of ASC. However, it significantly blocked the self‐oligomerization of NLRP3 in HEK293T cells (Figure 6G–N). These findings suggested that bigelovin disrupted the assembly of NLRP3 inflammasome by specifically targeting the oligomerization of NLRP3.

### 2.6 Bigelovin Directly Binds to the RACK1 Protein

**Figure 7:**
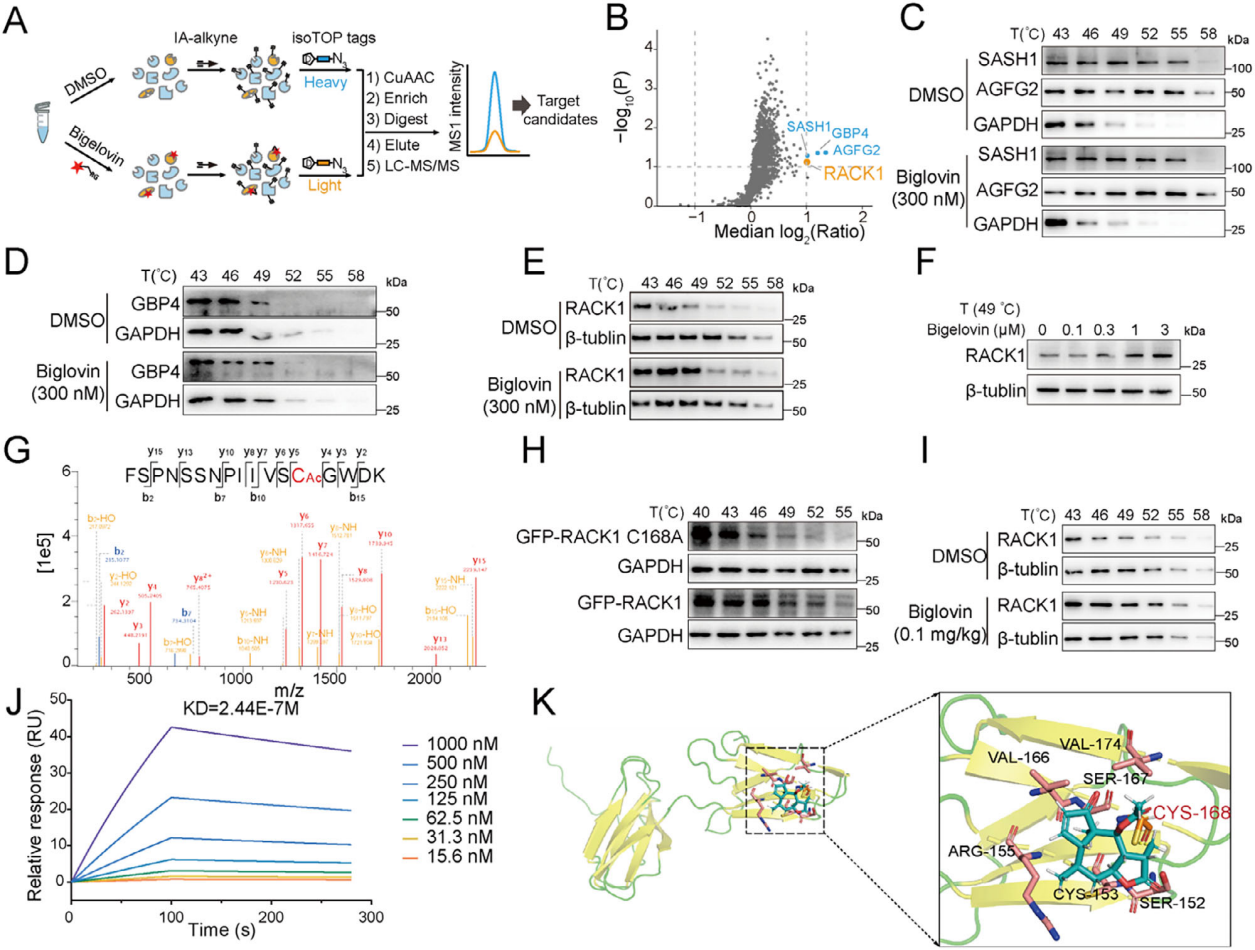
Bigelovin targets the RACK1 protein. A) Workflow of isoDTB‐ABPP experiment. B) Bigelovin binding proteins identified by isoDTB‐ABPP experiment. C–E) LPS‐primed BMDMs were incubated with DMSO or bigelovin (300 nM) for 1 h, and cellular thermal shift assays (CETSA) analyzed the thermal stabilization of Agfg2 (C), Sash1 (C), Gbp4 (D), RACK1 (E) at different temperatures (*n* = 3). F) LPS‐primed BMDMs were incubated with different concentration of bigelovin for 1 h, then thermal stability of RACK1 protein was evaluated by CESTA at 49 °C (*n* = 3). G) Mass spectrometry analysis of the binding site of bigelovin to RACK1. H) HEK‐293T cells transfected with indicated plasmid were incubated with bigelovin (300 nM) for 24 h, then thermal stability of GFP‐RACK1 and GFP‐RACK1 (C168A) protein were evaluated by CESTA at different temperature (*n* = 3). I) Mice were administrated with DMSO or bigelovin (0.1 mg kg^−1^) for 3 days by intraperitoneal injection and then challenged with 1% starch broth solution for 48 h, thermal stability of RACK1 protein in peritoneal macrophages was evaluated by CESTA at different temperature (*n* = 3). J) The interaction of bigelovin with RACK1 was measured by SPR. K) The interaction between bigelovin and RACK1 was measured by molecular docking.

Due to the presence of α,β‐unsaturated lactone/ketone in the structure of bigelovin, we suspect the compound may form a covalent bond with a potential target related to the process of NLRP3 assembly. To optimize the pull‐down tag placement, we evaluated the IL‐1β inhibition by structurally modified bigelovin analogs. The results showed a significant reduction in potency when the α,β‐unsaturated lactone at C_12_–C_14_ position, was reduced, implying a covalent inhibition at cysteine residue contained in the molecular target. Removal and substitution of the acetyl group at the C_6_ position also deteriorated the inhibitory activity (Figure S8A,B, Supporting Information). Unfortunately, the introduction of a click chemistry handle to compound 5 resulted in a more than tenfold drop in its activity (46 nM versus 627 nM, Figures S3C and S7C, Supporting Information), hindering the progress of our target identification efforts using the AfBPP method. Consequently, we chose the isoTOP‐ABPP analysis, a cysteine‐specific chemoproteomic approach for the target search (**Figure** **7**A). In the cysteinome of BMDM cells, we identified 1657 bigelovin‐modified cysteines presented across three biological replicates. Cysteines belonging to four proteins (RACK1, SASH1, GBP4, AGFG2) met the threshold (≥twofold ratio increase and *p*‐value ≤0.1) according to the literature (Figure 7B, raw data deposits in iProX with identifier PXD053000).^[^ ^20^ ^]^

To narrow the target range, we utilized CETSA to verify the interaction between bigelovin and specific proteins. The thermostability of SASH1, GBP4, and AGFG2 proteins remained unchanged in the presence of bigelovin (Figure 7C,D; Figure S9A–C, Supporting Information). In contrast, the RACK1 protein, which significantly degraded at 49 °C, exhibited increased thermostability when treated with bigelovin in BMDMs (Figure 7F; Figure S9E, Supporting Information). Strikingly, bigelovin maintained the stability of RACK1 in a dose‐dependent manner (Figure 7E; Figure S9D, Supporting Information). Mass spectrometry data confirmed that bigelovin directly bound to the Cys168 residue of RACK1. This was further substantiated by overexpressing WT‐RACK1 and the RACK1 (C168A) mutant in HEK293T cells, where bigelovin stabilized the WT‐RACK1 protein but not the mutant form (Figure 7G,H). Moreover, this binding was consistent in peritoneal macrophages from mice treated with bigelovin (0.1 mg kg^−1^), showing similar stabilization effects (Figure 7I; Figure S9F, Supporting Information). To quantify the interaction between bigelovin and RACK1, surface plasmon resonance (SPR) analysis determined a *K_D_* value of 0.24 µM (Figure 7J). Computational docking of bigelovin to RACK1 further supported the formation of a covalent bond at the Cys168 residue, in which surrounding residues (Ser152, Ser167, and Val166) may stabilize the molecule‐protein complex through non‐covalent interactions (Figure 7K).

### 2.7 Bigelovin Blocks NLRP3 Inflammasome Activation Mainly via Inhibition of RACK1

**Figure 8:**
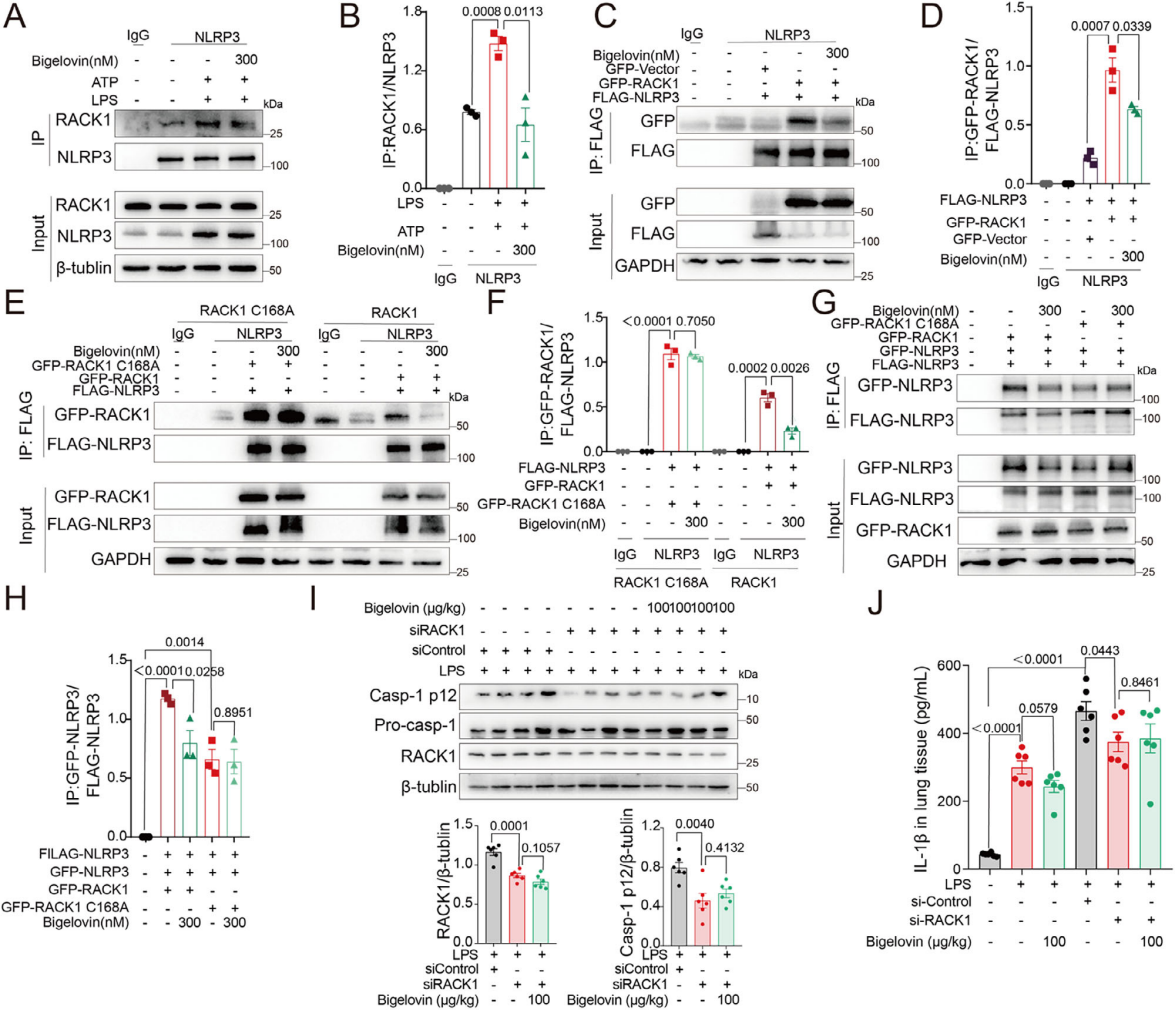
Bigelovin inhibits NLRP3 inflammasome by binding to cysteine 168 of RACK1. A,B) LPS‐primed BMDM was treated with bigelovin and then stimulated with ATP (5 mM, 30 min). Cell lysates were immunoprecipitated and immunoblotted with the indicated antibodies (A). Quantitative analysis of the interaction of NLRP3‐RACK1 described in (A) (*n* = 3) (B). C,D) HEK‐293T cells were transfected FLAG‐NLRP3 with GFP‐Vector or GFP‐RACK1 and treated with bigelovin (300 nM) for 24 h. Cell lysates were immunoprecipitated and immunoblotted for analysis of the interaction of FLAG‐NLRP3 and GFP‐RACK1 (C). Quantitative analysis of the interaction of GFP‐RACK1‐FLAG‐NLRP3 described in (C) (*n* = 3) (D). E,F) HEK‐293T cells were transiently transfected with and either GFP‐RACK1 or GFP‐RACK1 (C168A) and treated with bigelovin (300 nM) for 24 h. Samples were immunoprecipitated with FLAG and probed for FLAG (NLRP3) and GFP (RACK1 or RACK1 (C168A) (E). Quantitative analysis of the immunoblot of GFP‐RACK1/FLAG‐NLRP3 described in 9(E) (*n* = 3) (F). G,H) Immunoprecipitation analysis of NLRP3‐NLRP3 interaction in HEK‐293T cells transfected with FLAG‐NLRP3, GFP‐NLRP3, GFP‐RACK1 or GFP‐RACK1 mutant (C168A) plasmid and treated with bigelovin (300 nM) for 24 h (G). Quantitative analysis of the interaction GFP‐NLRP3‐FLAG‐NLRP3 described in (G) (*n* = 3) (H). I,J) Mice were intravenously injected with siRNA (80 µg per mouse) with in vivo‐jetPEI, followed by intraperitoneal injection of 7.5 mg kg^−1^ LPS 48 h later, and then injected intraperitoneally bigelovin for 12 h. I) Western blotting analysis of RACK1 and caspase‐1 (p12) from above mice (*n* = 6). J) ELISA quantification of IL‐1β levels of lung tissues from above mice were measured (*n* = 6). Data were presented as mean ± SEM and statistical significance was assessed by two‐tailed unpaired *t* test.

Considering RACK1's necessity for maintaining the active conformation of NLRP3,^[^ ^18^ ^]^ we hypothesized that bigelovin inactivated the NLRP3 inflammasome by targeting RACK1. Consistent with our hypothesis, bigelovin treatment prevented NLRP3 antibodies from precipitating RACK1 in BMDMs (**Figure** **8**A,B). A similar inhibitory result was observed in HEK293T cells using GFP and FLAG‐tagged proteins (Figure 8C,D). To determine whether the covalent binding of bigelovin to RACK1 was responsible for the inhibition of NLRP3 activation, wild‐type and C168A‐mutated RACK1 were co‐transfected with NLRP3 into HEK293T cells, respectively. Immunoprecipitation showed that while both forms of RACK1 interacted with NLRP3, the mutant RACK1‐NLRP3 interaction remained unaffected by bigelovin (Figure 8E,F). Moreover, bigelovin treatment blocked NLRP3 oligomerization mediated by wild‐type RACK1 but had a minimal impact on the NLRP3‐NLRP3 interaction mediated by mutant RACK1 (Figure 8G,H).

In the last experiment, we utilized an LPS‐induced ARDS model to elucidate the connection between RACK1 inhibition and NLRP3 inflammasome activation. By administering a siRNA cocktail via tail vein injection using the jetPET system, we observed that si‐*Rack1* treatment led to a decrease in RACK1 expression in the lungs, which correspondingly mitigated the severity of ARDS (Figure 8I,J; Figure S10A–D, Supporting Information). Specifically, si‐Rack1 remarkably suppressed the expression of activated caspase‐1 and the production of IL‐1β in the lung compared to si‐control groups, indicating inhibition of NLRP3 inflammasome activation. In contrast, the production of IL‐6 was not affected in all groups (Figure S10D, Supporting Information). Consistently, the secretion of IL‐1β was not altered by bigelovin treatment in the si‐Rack1 group but diminished by bigelovin treatment alone (Figure 8J; Figure S10C, Supporting Information). These findings collectively indicate that bigelovin acts as a covalent inhibitor of RACK1, thereby preventing the activation of the NLRP3 inflammasome mediated by RACK1 in vivo.

## 3 Discussion

ARDS is a prevalent and severe clinical condition that develops high mortality but lacks effective therapy in facing infectious pandemics such as COVID‐19.^[^ ^27^ ^]^ Recent studies, including our work herein, have recognized the abnormal accumulations of IL‐1β and its producer NLRP3 inflammasome in the collected samples from ARDS patients.^[^ ^28^, ^29^ ^]^ Although antagonistic therapies of IL‐1β and its receptor have been entered the clinic for decades, the overall efficacy could be compromised because multiple pro‐inflammatory mediators except IL‑1β could be over‐produced by NLRP3.^[^ ^30^, ^31^ ^]^ On the other hand, side effects such as high risks of upper respiratory infection and pneumonia are common in the patients administrated with IL‐1β antagonists. Thus, pharmacological inhibition of NLRP3 inflammasome activation may provide potent therapeutic effects in diseases with over‐inflammatory responses, including ARDS. Several heterocyclic candidates including MCC950^[^ ^32^ ^]^ and OLT1177,^[^ ^33^ ^]^ as well as a series of phytochemicals such as oridonin,^[^ ^34^ ^]^ costunolide,^[^ ^35^ ^]^ arglabin,^[^ ^36^ ^]^ were found directly targeting NLRP3. For example, MCC950 directly binds to the Walker B motif within the NACHT domain and blocks ATP hydrolysis,^[^ ^32^ ^]^ OLT1177 also directly targets NLRP3 NACHT domain and inhibits its ATPase activity; while oridonin covalently binds with the cysteine 279 in NACHT domain of NLRP3 to inhibit the binding of NLRP3 and NEK7, thereby blocking its assembly and activation. However, the clinical advancement of MCC950 and OLT1177 has been suspended, due to the unexpected side‐effect^[^ ^37^ ^]^ and less efficacy in phase 2 trials,^[^ ^38^ ^]^ respectively. The absence of approved drugs casts doubt on the viability of strategies that target NLRP3 directly.

The conserved α‐methylene‐γ‐lactone moiety among NLRP3‐inhibiting phytochemicals suggested a structure‐activity relationship, motivating systematic evaluation of sesquiterpene lactone libraries.^[^ ^35^ ^]^ *Inula helianthus aquatica*, known to enrich such a structure, was identified as the source of major component in TCM decoctions used to treat respiratory disorders such as bronchitis and asthma.^[^ ^23^ ^]^ In vivo models of ARDS demonstrated that three sesquiterpene lactones found in the plant, bigelovin, ergolide, and 8‐epi‐helenalin, showed potent preventive and therapeutic effects. Although previous research has reported their anti‐inflammation activity, the mechanisms remained uncertain.^[^ ^39^, ^40^ ^]^ Our research here defined that these compounds inhibit NLRP3 inflammasome activation at nanomolar concentrations, with bigelovin showing the most potent activity in both mouse and human macrophages.

According to our results, bigelovin selectively inhibits various forms of NLRP3 inflammasome activation—canonical, noncanonical, and alternative—without impacting other inflammasomes like AIM2 and NLRP1. Two salient insights regarding its anti‐inflammatory mechanism featured further notes. (i) The anti‐inflammatory activity of bigelovin is concentration‐dependent. Figures 2 and 4 illustrate that the concentration required to inhibit NLRP3 inflammasome activation and the subsequent release of IL‐1β is significantly lower than that affecting IL‐6 and TNF‐α production, which is mediated transcriptionally via the NF‐κB pathway. This aligns with prior findings that bigelovin suppresses NF‐κB activation and pro‐inflammatory cytokine production starting at a concentration of 2 µM.^[^ ^23^ ^]^ Notably, our in vivo model also verified that IL‐1β levels were significantly lower than IL‐6 at equivalent dosages (Figure 4). (ii) The administration method and timing influence the therapeutic outcomes in the ARDS mice. While oral administration yielded positive results, pre‐LPS challenge injection proved to be the optimal approach for acute inflammation, indicating that the bioavailability of bigelovin is unsatisfactory. Consequently, we infer that administering a low dosage of bigelovin via injection or employing advanced pharmaceutical technologies to target lung tissue, could amplify its therapeutic benefits and minimize side effects in treating NLRP3‐related inflammatory lung conditions. This will be the focus of our subsequent lead optimization efforts.

In addition, the upstream events in the process of NLRP3 activation, such as K^+^ efflux or Ca^2+^ influx, mitochondrial damage, and lysosome damage were not intervened by bigelovin treatment. Importantly, our results showed that bigelovin could block the interaction of NLRP3 and NEK7 as well as the interaction of NLRP3 and ASC, and the oligomerization of NLRP3 and the assembly of ASC specks, which are indispensable for NLRP3 inflammasome assembly and activation. Subsequently, we determined that bigelovin inhibited the self‐association of NLRP3 by conducting the overexpression experiment in HEK293T cells, revealing that NLRP3 activation mainly through regulation of NLRP3 oligomerization at low concentrations. During the activation of the NLRP3 inflammasome, the NACHT domain of NLRP3 plays a pivotal role in its oligomerization and subsequent activation. Although recent cryo‐electron microscopy structure studies revealed that NLRP3 was segregated into two distinct oligomeric states, the smaller complex largely lacks activity. There is no doubt that PAMPs or DAMPs stimuli induce NLRP3 from a closed, auto‐inhibited conformation to an active “open” conformation that coincides with its oligomerization.^[^ ^41^ ^]^

Cryo‐electron microscopy has also revealed that the NACHT domain of NLRP3, also known as NOD, transitions from a closed, auto‐inhibited, ADP‐bound inactive state to an open, ATP‐bound active state before its oligomerization and subsequent activation.^[^ ^9^ ^]^ NEK7 was successfully identified as a chaperone inserted into the leucine‐rich repeat (LRR) domain, releasing the nucleotide‐binding domain (NBD) and facilitating nucleotide exchange. However, NEK7 alone is deficient in conducting such large conformational changes, as ADP remains bound to the NBD in the NLRP3‐NEK7 complex.^[^ ^41^ ^]^ RACK1 was discovered to be another binding partner interacting with the NLRP3‐NEK7 complex at the NACHT region, independently of its activated protein kinase C (PKC) kinase activity. Although further structural insights into the conformational changes are required, knockdown and bioluminescence resonance energy transfer (BRET) experiments have helped delineate the conformational transition of NLRP3 in response to RACK1. Our study illustrates that bigelovin acts as a Michael receptor, forming a covalent bond with Cys168 on RACK1, which significantly inhibits its interaction with NLRP3, thereby hindering activation. Wild‐type or mutant RACK1 revealed that cysteine 168 is the key residue in the interaction between RACK1 and NLRP3, providing structural insight into how RACK1 mediated the self‐interaction of NLRP3. We also sought to corroborate the mechanism of biglovin in the ARDS mouse model. Even though the complete deletion of RACK1 is lethal for early embryonic development, treatment with siRNA showed that *Rack1* knockdown remarkably attenuated caspase‐1 activation and IL‐1β secretion in the lung tissue, whereas the production of IL‐6 was less affected. Following our finding, the secretion of IL‐1β in alveolar macrophages in *vivo* was no longer altered by bigelovin administration in the *Rack1* knockdown group, which validated the molecular mechanism of RACK1 and NLRP3 activation as well as the accuracy of cysteinomic ABPP method in the target identification. Indeed, tail vein injection of siRNA by the jetPET system is generalized to reduce target protein. While our data demonstrate RACK1's role in alveolar macrophages, we cannot exclude potential anti‐inflammatory contributions from RACK1 inhibition in other cell types.

Finally, covalent inhibitors have been confronted with safety concerns due to the presence of Michael acceptor. However, bigelovin only tackled four different proteins with statistical significance in the cysteinome of BMDMs, illustrating the structure may be stable to most bioactive cysteine residues in the concentration range effective to Rack1. The in vivo study also proved that bigelovin exhibited no toxicity at a 100‐fold dose (10 mg kg^−1^, i.g.) compared to its positive dose in mice (0.1 mg kg^−1^, i.g.).

## 4 Conclusions

In summary, our study reported that bigelovin was covalently bound to the cysteine 168 of RACK1 and inhibited the interaction between RACK1 and NLRP3, thereby interfering with the activation of NLRP3 inflammasome in vitro and in vivo. Bigelovin showed significant anti‐inflammasome efficacy in NLRP3‐related pulmonary disorders murine models of LPS‐induced ARDS and silicosis by inhibiting NLRP3 inflammasome activation. These findings established the complex involvement of RACK1 in NLRP3 state transition and offered a novel anti‐inflammatory approach and treatment for NLRP3‐related illnesses.

## 5 Experimental Section

### Animals

C57BL/6J mice (male, 6–8 weeks) were purchased from Gempharmatech Co., Ltd. (Nanjing, Jiangsu, China). All mice experiments were conducted following the Guide for the Care and Use of Laboratory Animals (Ministry of Science and Technology of China, 2006) and were approved by the Animal Ethics Committee of Nanjing University of Chinese Medicine (NO. 202210A005). Mice were housed in Experimental Animal Center of Nanjing University of Chinese Medicine at 23–26 °C and humidity 40–60% for 7 days, with 12 h dark/night cycle and free access to food and water.

### Cell Culture

Human Peripheral Blood Mononuclear Cells (PBMCs) were isolated from healthy volunteers. This study was approved by the Institutional Research Ethics Committee of Jiangsu Provincial Hospital of Chinese Medicine (Approved Number: 2021NL‐095‐02). Bone marrow‐derived macrophages (BMDMs) were cultured in RPMI 1640 medium supplemented with 20% L929 supernatant. PBMCs, L929 cells (ATCC), THP‐1 cells (ATCC) were cultured in RPMI 1640 and HEK293T cells (ATCC) were cultured in DMEM, all medium were supplemented with 10% fetal bovine serum, and 1% Penicillin/Streptomycin. And cells were cultured in a constant humidity incubator with 5% CO_2_ at 37 °C.

### Antibodies and Reagents

Anti‐NLRP3 (Adipogen, AG‐20B‐0014‐c100), Anti‐Caspase‐1+p10+p12 (Abcam, ab179515), Anti‐IL‐1β+p17 (Abcam, ab234437), Anti‐Asc (Adipogen, AG‐25B‐0006‐c100), Anti‐Phospho‐NF‐kappaB p65 (Abmart, TP56372F), Anti‐NF‐кB p65 (Abmart, T55034F), Anti‐β‐actin (Abmart, T401045), Anti‐GAPDH (Abmart, P60037), Anti‐β‐tublin (Abmart, M20005), Anti‐FLAG (Abmart, M20008), Anti‐HA (Abmart, M20003), Anti‐GFP (Santa Cruz, sc‐9996), Anti‐RACK1 (Huabio, ET7109‐04), Anti‐SASH1 (Bioss, bs‐6099R), Anti‐AGFG2 (UpingBio, YP‐Ab‐04108), Anti‐GBP4 (Abmart, PA5012), Anti‐FITC‐ly6G (Biolegend, 127 605), Anti‐APC‐F4/80 (Biolegend, 123 116), Anti‐Collagen Type I (Proteintech, 14695‐1‐AP), Anti‐Smooth muscle actin (Proteintech, 14395‐1‐AP), Goat anti‐mouse IgG (Abbkine, A21010), Goat anti‐rabbit IgG (Abbkine, A21020). Recommended concentrations were used for all antibodies.

Lipopolysaccharides (LPS, Sigma, L4391), Adenosine triphosphate (ATP, Aladdin, A100885), Uric acid sodium salt (MSU, Sigma, U2B75), Nigericin (Shanghai Yuanye Bio‐Technology, S25116), Pam3CSK4 (Invitrogen, tlrl‐pms), Anisomycin (MedChemExpress, HY‐18982), Poly(dA:dT) (Invitrogen, tlrl‐patn), PMA (Sigma, P1585), MCC950 (TargetMOI, XSD20220316‐00020), Dexamethasone (Dex, Sigma, D4902), Nintedanib (Meilunbio, MB7360).

### Chemistry

The dried flowers of *Inula helianthus aquatilis* C. Y. Wu ex Y. Ling were added to 10 times the amount of 70% ethanol and cold‐soaked for 2 h, followed by three rounds of reflux extraction for 2 h each. The filtrates were then combined and subjected to column chromatography using D101 macroporous resin with a gradient elution of 30%–90% ethanol. The components were tracked by HPLC, enriching the sesquiterpene lactone fraction, which was then quantified for subsequent activity evaluation.

The dried flowers of *Inula helianthus aquatilis* C. Y. Wu ex Y. Ling (300 g) were extracted under reflux with petroleum ether (PE) (3 L × 2 h, three times). After removal of the PE in vacuo, the combined extract (15 g) was subjected to silica gel column chromatography (PE/EA, 200:1→2:1), monitored by thin layer chromatography. The extract was chromatographically separated on a silica gel column and eluted with PE‐EA to obtain ergolide (430 mg), bigelovin (153 mg), and 8‐epi‐helenalin (150 mg).

Single‐cell RNA Sequencing (scRNA‐seq) analysis

### PBMC Sample

A dataset of scRNA‐seq (GSE175450) was collected from the Tumor Immune Single‐cell Hub (TISCH) database.^[^ ^24^ ^]^ The standard workflow for processing scRNA‐seq data was performed using the R package “Seurat v4”.^[^ ^42^ ^]^ We used the Uniform Manifold Approximation and Projection (UMAP) coordinates and annotation information provided by TISCH and visualized them with the “plot1cell” package.^[^ ^43^ ^]^ The expression of genes and gene signatures was described by the “scRNAtoolVis” package.^[^ ^44^ ^]^

### BALF Sample

Using cell type‐specific marker gene expression analysis, cell types were distinguished from the scRNA‐seq data. The R packages “celldex” and “SingleR” were used to classify the immune cell populations, including but not limited to T cells, B cells, natural killer cells, dendritic cells, and monocytes. Each immune cell type was identified based on its known marker genes and characterized by its distinct gene expression signature using the “MonacoImmuneData” reference index, which contains normalized expression values of 114 bulk RNA‐seq samples derived from sorted immune cell populations, enabling high‐resolution profiling of immune cell transcriptome.^[^ ^45^ ^]^

### Serum Samples from ARDS Patients

Whole blood samples were collected from ARDS patients and centrifuged at 3,000 rpm for 15 min at 4 °C to obtain serum. This study was approved by the Institutional Research Ethics Committee of Jiangsu Provincial Hospital of Chinese Medicine (Approved Number: 2021NL‐095‐02).

### Inflammasome Stimulation ^[^ ^46^ ^]^

Canonical NLRP3 inflammasome was activated as follows: BMDMs were primed with LPS (100 ng mL^−1^) for 3 h and treated with bigelovin for 1 h, followed by ATP (5 mM) for 45 min, nigericin (5 µM) for 45 min, and MSU (500 µg mL^−1^) for 12 h. For noncanonical NLRP3 activation, the cells were primed with Pam3CSK4 (500 ng mL^−1^) for 3 h and treated with bigelovin for 1 h. After that, LPS (2 µg) was transfected into BMDMs using Lipofectamine 3000 (Invitrogen, L3000015) for 24 h. For alternative NLRP3 activation, PBMCs were treated with bigelovin for 1 h and then stimulated with LPS (100 ng mL^−1^) for 16 h.

Other inflammasomes were stimulated as follows: For NLRP1 inflammasome activation, BMDMs were primed with LPS (100 ng mL^−1^) for 3 h and treated with bigelovin for 1 h. Subsequently, the cells were stimulated with anisomycin (1 µM) for 24 h.^[^ ^47^ ^]^ AIM2 inflammasome activation was obtained by transfection of 2 µg poly(dA:dT) using Lipofectamine 3000 (Invitrogen, L3000015) for 6 h.

THP‐1 cells were primed with PMA (500 nM) for 3 h and then treated with bigelovin for 1 h after stimulated with LPS (100 ng mL^−1^) for 3 h. Subsequently, the cells were stimulated with ATP (5 mM) for 45 min.

### Enzyme‐linked immunosorbent assay (ELISA)

The ELISA experiments procedure has been described previously.^[^ ^19^ ^]^

### Cell counting kit 8 (CCK‐8)

The CCK‐8 assay has been described previously.^[^ ^19^ ^]^

### Lactate dehydrogenase assay (LDH)

BMDMs were seeded in 24‐well plates overnight and stimulated with 100 ng mL^−1^ LPS for 3 h, treated with bigelovin at the indicated concentrations for 1 h. After that, stimulated with 5 mM ATP for 45 min, LDH release was detected by the LDH Cytotoxicity Assay Kit (Beyotime, C0016) following the manufacturer's instructions.

### Western blot and co‐immunoprecipitation

The protocols for immunoprecipitation and co‐immunoprecipitation assay have been described previously.^[^ ^48^, ^49^ ^]^

### Quantitative real‐time PCR (qRT‐PCR)

The protocol for qRT‐PCR has been described previously.^[^ ^48^ ^]^ The primer sequences used in the study were described below:

*Gapdh*MusFor: CATCACTGCCACCCAGAAGACTG

*Gapdh*MusRev: ATGCCAGTGAGCTICCCGITCAG

*Il1b*MusFor: TGGACCTTCCAGGATGAGGACA

*Il1b*MusRev: GTTCATCTCGGAGCCTGTAGTG

*Il6*MusFor: TACCACTTCACAAGTCGGAGGC

*Il6*Mus Rev: CTGCAAGTGCATCATCGTIGTTC

*Tnfa*MusFor: CGTGCCTATGTCTCAGCCTCTT

*Tnfa*MusRev: GCCATAGAACTGATGAGAGGGAG

*Rack1*MusFor: TCCTCTGATGGTCAGTTTGCCC

*Rack1*MusRev: CACGCTCAACACATCCTTGGTG

*ASC oligomerization assay*: The assay for ASC oligomerization has been described previously.^[^ ^19^, ^50^ ^]^

### NLRP3 Oligomerization Assay

BMDMs were stimulated by ATP (5 mM) as described above. The cells were collected and resuspended in HEPES, subjected to 20 strokes of homogenization using a syringe, followed by centrifugation at 4 °C, 900 ×*g* for 8 min to remove cell nucleus and unbroken cells. After centrifugation at 6200 ×*g* for 8 min, supernatants were discarded and the pellets were resuspended in 200 µL of HEPES. For cross‐linking, 2 mM DSS was added and incubated at room temperature for 1 h. The samples were then dissolved in sample buffer, and NLRP3 oligomerization was detected by western blotting.

### Intracellular Ca^2+^ and K^+^ Measurement

BMDMs after stimulation were washed with PBS. After digestion by pancreatic enzymes, the cells were centrifuged and washed again. Subsequently, BMDMs were incubated with Fluo‐4 AM (2 µM) (Beyotime, S1060) for 30 min at 37 °C. After centrifugation, the samples were resuspended in 300 µL PBS and incubated for 30 min at 37 °C once more and subsequently analyzed by flow cytometry on Beckman Coulter Gallios.

BMDMs were incubated with ION Potassium Green‐2 AM (10 µM) (Abcam, ab142806) for 15 min at 37 °C and then stimulated with 5 mM ATP for 45 min. The levels of intracellular K^+^ were determined by flow cytometry on Beckman Coulter Gallios.

### Measurement of Lysosome Rupture

BMDMs after stimulation were washed with RPMI 1640 medium once. The cells were loaded with Lyso‐Tracker Red (1 µM) (Solarbio, L8010) for 30 min at 37 °C. After that, the cells were digested by pancreatic enzymes before centrifugation and washed with PBS. The samples were analyzed by flow cytometry after resuspending in 200 µL PBS.

### Measurement of mtROS

BMDMs were stimulated by ATP as previously described. Then, the cells were washed twice with PBS and incubated with DCFH‐DA (10 µM) (Beyotime, S0033) for 30 min at 37 °C. After centrifugation, the samples were resuspended in 200 µL PBS after rinsing and subsequently analyzed by flow cytometry.

### Confocal Microscopy

BMDMs after stimulation were incubated with Mito‐Tracker Red CMXRos (200 nM) (Beyotime, C0135) for 30 min before sample collection. The cells were washed twice with PBS and fixed with 4% PFA for 30 min at room temperature. Then, the cells were washed three times by PBST and stained with DAPI (Beyotime, C1005) for 10 min. Subsequently, the cells were washed three times by PBST again. Confocal microscopy analysis was analyzed by fluorescence microscope on Lecia TCS SP8.

### Plasmid Transfection

Plasmids of FLAG‐NLRP3, GFP‐NLRP3, FLAG‐NEK7, FLAG‐ASC, GFP‐ASC, GFP‐Vector, GFP‐RACK1, GFP‐RACK1 C168A were manufactured by General Biotechnology (Hefei, China). DNA of plasmids were transfected to HEK293T using Lipofectamine 3000 for 24 h, then cells were treated with bigelovin for another 24 h for co‐immunoprecipitation assays.

### IsoTOP‐ABPP Cysteine Chemoproteomic Profiling

The BMDMs were treated with (100 ng mL^−1^) LPS for 3 h, then 1 µM bigelovin or the corresponding concentration of the DMSO was added. Cells steady at 37 °C for 1 h before lysis with PBS. Protein concentration was determined with BCA assay and the concentration was adjusted to 1 µg µL^−1^ with PBS. For biological replicates, two aliquots of 1 mL cell lysates were prepared. Each aliquot was treated with 100 µM IA‐alkyne at room temperature (RT) for 1 h The click reaction reagent containing 60 µL 0.9 mg mL^−1^ TBTA (in 4:1 tBuOH/DMSO), 20 µL 12.5 mg mL^−1^ CuSO_4_ (in H_2_O), 20 µL 13 mg mL^−1^ TCEP (in H_2_O) and 20 µL 5 mM light isoDTB tags (DMSO) or heavy isoDTB tags (bigelovin) were prepared. Samples were treated with 120 µL click reaction reagent at RT for 1 h. After incubation, the light and heavy labeled samples were combined and precipitated with cold acetone. Protein precipitates were washed with methanol and dissolved followed by enrichment with streptavidin agarose beads. After reduction and alkylation, on‐bead digestion was performed with 10 ng µL^−1^ trypsin at 37 °C overnight. The beads were washed and then peptides attached to the beads were eluted with 0.1% formic acid in 50% acetonitrile in water. Peptides were analyzed on a Q Exactive Plus with an EASY‐nLC 1200 system. Samples were separated at a flow rate of 300 nL min^−1^ using the following gradient: 2% to 5% buffer B (80% acetonitrile with 0.1% formic acid in H_2_O) in buffer A (0.1% formic acid in H_2_O) for 2 min, followed by a gradient from 5%–32% B for 76 min, 32%–45% buffer B for 5 min, 45%–100% B for 2 min and holding at 100% B for 2 min. Column temperature was maintained at 50 °C. The scan range of MS1 was 300–1700 m z^−1^ with a resolution of 70 000 (at 200 m z^−1^), with an AGC target of 3×10^6^ and a maximum injection time of 50 ms. The top 20 precursors were selected for MS/MS analysis with a resolution at 17 500 (at 200 m z^−1^), AGC target 1×10^5^, and a maximum injection time of 100 ms. The isolation window of precursors was 2 m z^−1^. Normalized collision energy was set at 27 eV with a 30 s dynamic exclusion window. Data analysis for the isoTOP‐ABPP assay was carried out as described previously.^[^ ^20^, ^49^ ^]^

### Cellular Thermal Shift Assay

The BMDMs were incubated with bigelovin (300 µM) or DMSO for 1 h. Harvested cells were frozen and thawed three times by liquid nitrogen. The proteins were separated from cells by centrifuging at 15,000 *g* for 10 min at 4 °C. Subsequently, the supernatant was equally divided into 6 parts, heated for 3 min at different temperatures, and detected protein level by immunoblotting.

C57BL/6 mice were injected intraperitoneally with bigelovin (0.1 mg kg^−1^) for three consecutive days, and then peritoneal macrophages were harvested to CETSA in vivo.

### Surface Plasmon Resonance

The binding affinity between bigelovin and recombinant human RACK1 protein (Biorbyt, orb754920) was assayed using a WeSPR One instrument. Bigelovin was loaded to the CM5 sensor chip (LifeDisc, G70015). The concentration gradient RACK1 was prepared with PBS running buffer (pH = 7.4, 0.05% Tween‐20), and flowed over the chip. The parameters of SPR used in the study were described below: flow rate, 20 µL min^−1^; temperature, 22 °C; association time, 100 s; disassociation time, 180 s. The equilibrium dissociation constant (*K_D_*) was calculated using WeSPRone Auto software, version 5.3.0, developed by Xlement.^[^ ^51^ ^]^

### Molecular Docking

The protein structure of RACK1 (UniProt ID: D6RBD0) was derived from the AlphaFold Protein Structure Database (https://alphafold.ebi.ac.uk/) and prepared by Schrödinger 2019 protein wizard module. The amino acid sequence in D6RBD0 was renumbered based on the sequence utilized in the constructed plasmid transfected into the cells. Bigelovin was processed using the LigPrep module and docked using the covalent dock module in the Schrödinger 2019. During the setup process, the reaction type was selected as Michael addition and the scoring function was set to Extra Precision.^[^ ^52^ ^]^

### LPS‐Induced Mice

To assess the preventive effect, C57BL/6J mice were injected intraperitoneally fractions (0.1 or 1 mg kg^−1^), bigelovin (10 or 100 µg kg^−1^), vehicle, dexamethasone (5 mg kg^−1^) for three consecutive days. 30 min after the last dose, 7.5 mg kg^−1^ LPS (Sigma, L2630) was injected intraperitoneally. The serum, BALF and lung tissues were collected after 12 h, and inflammatory cytokines were measured by qRT‐PCR or ELISA. The number of cells in the BALF was counted, and the number of macrophages (F4/80^+^ cells) and neutrophils (Ly6G^+^ cells) in the BALF were analyzed by flow cytometry. Lung samples were collected 24 h after LPS administration and fixed in 4% paraformaldehyde for histopathological evaluation. The prophylactic effect of oral bigelovin (0.1 or 1 mg kg^−1^) administration was evaluated as described above. To study the therapeutic effect, the mice were injected intraperitoneally 7.5 mg kg^−1^ LPS. After 30 min, the mice were injected intraperitoneally fractions (0.1 or 1 mg kg^−1^), bigelovin (10 or 100 µg kg^−1^), vehicle, and dexamethasone (5 mg kg^−1^). The mice were euthanized 24 h later. At the experimental endpoint, serum and lungs were collected for detection of mRNA, protein, or pathological damage.

### Biosafety Evaluation

C57BL/6J mice were intragastric administration of bigelovin (1 mg kg^−1^, 10 mg kg^−1^) for thirty consecutive days. The weight and overall condition of the mice were monitored throughout the experiment. On day 30, the mice were euthanized, and liver, heart, spleen, lung, kidney, and colon tissues were collected for subsequent analysis.^[^ ^18^ ^]^

### RACK1 Knockdown In Vivo

The siRNAs (si*Control*: 5´‐UUCUCCGAACGUGUCACGUTT‐3´; si*RACK1*: 5´‐GUAGAUGAAUUGAAGCAAGTT‐3´) were synthesized by General Biotechnology (Hefei, China). C57BL/6J mice were intravenously injected with siRNA (80 µg per mouse) using in vivo‐jetPEI (Polyplus, 101 000 030), followed by intraperitoneal injection of 7.5 mg kg^−1^ LPS 48 h later, and then the mice were injected intraperitoneally bigelovin for 12 h. The lung tissues were collected for the further assay.^[^ ^53^ ^]^

### SiO_2_‐Induced Mice

C57BL/6J mice were randomly divided into five groups, including normal, model, bigelovin (intraperitoneal injection of 0.1 mg kg^−1^, 1 mg kg^−1^), and positive control group treated with nintedanib (intragastric administration of 100 mg kg^−1^). The SiO_2_ (Sigma, S5631) suspension (300 mg kg^−1^) was injected into the trachea of mice, and the control group was injected with physiological saline. 7 days after SiO_2_ treatment, mice were injected intraperitoneally with bigelovin or given nintedanib by gavage for 14 consecutive days. On day 21, the mice were euthanized for subsequent analysis.

### DSS‐Induced Colitis

Colitis in mice was induced by 2.5% DSS in drinking water for 7 consecutive days followed by a 2‐day tapwater period, according to previous research.^[^ ^49^ ^]^ The bigelovin at the dose of 0.01, 0.1 mg kg^−1^ was given intraperitoneally once a day for 7 days during DSS administration. The solvent was given intraperitoneally to both sham and model groups as vehicle control.

### Immunohistochemistry

Formalin‐fixed, paraffin‐embedded tissues from mice were stained with Anti‐Collagen Type I antibody and Anti‐Smooth muscle actin as previously described.^[^ ^49^ ^]^

### Statistical Analysis

Statistical analyses were performed using GraphPad Prism 8.0.1. All data were analyzed by two‐tailed *t*‐tests or one‐way ANOVA (clinical samples) as appropriate, and *p* < 0.05 was considered statistically significant.

## Conflict of Interest

The authors declare no conflict of interest.

## Author Contributions

J.C., M.Y., C.Y. contributed equally to this work. J.C., M.Y. and L.H. conceived the research; J.C., M.Y., C.Y., Y.Z. and L.H. designed the methodology; J.C. and M.Y., Y.G., A.P., Q.Y., X.P. and A.W. performed the experiments; J.C., M.Y., Y.Z. wrote the original draft of the manuscript; J.C., Y.Z. and L.H. reviewed and edited the manuscript; J.C., Y.Z. and L.H. were involved in acquiring funding; J.C., H.Z., Y.H., Y.H., and Q.W. were involved in obtaining resources; Y.W. and J.L. were involved in single cell sequencing analysis, Z.J. contributed to molecular docking, J.C., Y.Z. and L.H. supervised the study. All authors gave final approval of the submitted and published versions of the manuscript.

## Supporting information



Supporting Information

Supporting Information

## Data Availability

The data that support the findings of this study are openly available in none at DOI, reference number 0.

## References

[1] N. J. Meyer , L. Gattinoni , C. S. Calfee , Lancet 2021, 398, 622. 34217425 10.1016/S0140-6736(21)00439-6 PMC8248927

[2] L. Bos , L. B. Ware , Lancet 2022, 400, 1145. 36070787 10.1016/S0140-6736(22)01485-4

[3] M. A. Matthay , R. L. Zemans , G. A. Zimmerman , Y. M. Arabi , J. R. Beitler , A. Mercat , M. Herridge , A. G. Randolph , C. S. Calfee , Nat. Rev. Dis. Primers 2019, 5, 18. 30872586 10.1038/s41572-019-0069-0 PMC6709677

[4] N. Sinha , A. K. Thakur , Trends Microbiol. 2021, 29, 967. 33795156 10.1016/j.tim.2021.03.008 PMC8007089

[5] S. Nye , R. J. Whitley , M. Kong , Front. Pediatr. 2016, 4, 128. 27933286 10.3389/fped.2016.00128 PMC5121220

[6] K. Peukert , M. Fox , S. Schulz , C. Feuerborn , S. Frede , C. Putensen , H. Wrigge , B. M. Kummerer , S. David , B. Seeliger , T. Welte , E. Latz , D. Klinman , C. Wilhelm , F. Steinhagen , C. Bode , Am J. Respir. Crit. Care Med. 2021, 204, 53. 33760701 10.1164/rccm.202005-1916OC PMC8437127

[7] A. C. Ferreira , V. C. Soares , I. G. de Azevedo‐Quintanilha , S. Dias , N. Fintelman‐Rodrigues , C. Q. Sacramento , M. Mattos , C. S. de Freitas , J. R. Temerozo , L. Teixeira , H. E. Damaceno , E. A. Barreto , C. Pao , L. Palhinha , M. Miranda , D. C. Bou‐Habib , F. A. Bozza , P. T. Bozza , T. Souza , Cell Death Discovery 2021, 7, 43. 33649297 10.1038/s41420-021-00428-w PMC7919254

[8] Q. Ma , Pharmacol. Rev. 2023, 75, 487. 36669831 10.1124/pharmrev.122.000629 PMC10121800

[9] L. Xiao , V. G. Magupalli , H. Wu , Nature 2023, 613, 595. 36442502 10.1038/s41586-022-05570-8 PMC10091861

[10] T. Tang , X. Lang , C. Xu , X. Wang , T. Gong , Y. Yang , J. Cui , L. Bai , J. Wang , W. Jiang , R. Zhou , Nat. Commun. 2017, 8, 202. 28779175 10.1038/s41467-017-00227-x PMC5544706

[11] G. S. Lee , N. Subramanian , A. I. Kim , I. Aksentijevich , R. Goldbach‐Mansky , D. B. Sacks , R. N. Germain , D. L. Kastner , J. J. Chae , Nature 2012, 492, 123. 23143333 10.1038/nature11588 PMC4175565

[12] A. Akbal , A. Dernst , M. Lovotti , M. Mangan , R. M. Mcmanus , E. Latz , Cell Mol. Immunol. 2022, 19, 1201. 36127465 10.1038/s41423-022-00922-w PMC9622870

[13] C. Guo , Z. Chi , D. Jiang , T. Xu , W. Yu , Z. Wang , S. Chen , L. Zhang , Q. Liu , X. Guo , X. Zhang , W. Li , L. Lu , Y. Wu , B. L. Song , D. Wang , Immunity 2018, 49, 842. 30366764 10.1016/j.immuni.2018.08.021

[14] Z. Zhang , G. Meszaros , W. T. He , Y. Xu , M. H. de Fatima , L. Mailly , M. Mihlan , Y. Liu , G. M. Puig , A. Goginashvili , A. Pasquier , O. Bielska , B. Neven , P. Quartier , R. Aebersold , T. F. Baumert , P. Georgel , J. Han , R. Ricci , J. Exp. Med. 2017, 214, 2671. 28716882 10.1084/jem.20162040 PMC5584123

[15] V. G. Magupalli , R. Negro , Y. Tian , A. V. Hauenstein , G. Di Caprio , W. Skillern , Q. Deng , P. Orning , H. B. Alam , Z. Maliga , H. Sharif , J. J. Hu , C. L. Evavold , J. C. Kagan , F. I. Schmidt , K. A. Fitzgerald , T. Kirchhausen , Y. Li , H. Wu , Science 2020, 369, aas8995. 10.1126/science.aas8995 PMC7814939 32943500

[16] Y. He , M. Y. Zeng , D. Yang , B. Motro , G. Nunez , Nature 2016, 530, 354. 26814970 10.1038/nature16959 PMC4810788

[17] K. Nozaki , E. A. Miao , Nature 2019, 570, 316. 31213677 10.1038/d41586-019-01764-9

[18] Y. Duan , L. Zhang , D. Angosto‐Bazarra , P. Pelegrin , G. Nunez , Y. He , Cell Rep. 2020, 33, 108405. 33207200 10.1016/j.celrep.2020.108405 PMC7709964

[19] Q. Lv , Y. Xing , J. Liu , D. Dong , Y. Liu , H. Qiao , Y. Zhang , L. Hu , Acta Pharm. Sin. B 2021, 11, 2880. 34589402 10.1016/j.apsb.2021.03.011 PMC8463273

[20] P. Zanon , L. Lewald , S. M. Hacker , Angew. Chem., Int. Ed. Engl. 2020, 59, 2829. 31782878 10.1002/anie.201912075 PMC7027453

[21] C. Huang , Y. Wang , X. Li , L. Ren , J. Zhao , Y. Hu , L. Zhang , G. Fan , J. Xu , X. Gu , Z. Cheng , T. Yu , J. Xia , Y. Wei , W. Wu , X. Xie , W. Yin , H. Li , M. Liu , Y. Xiao , H. Gao , L. Guo , J. Xie , G. Wang , R. Jiang , Z. Gao , Q. Jin , J. Wang , B. Cao , Lancet 2020, 395, 497. 31986264 10.1016/S0140-6736(20)30183-5 PMC7159299

[22] M. Lyu , G. Fan , G. Xiao , T. Wang , D. Xu , J. Gao , S. Ge , Q. Li , Y. Ma , H. Zhang , J. Wang , Y. Cui , J. Zhang , Y. Zhu , B. Zhang , Acta Pharm. Sin. B 2021, 11, 3337. 34567957 10.1016/j.apsb.2021.09.008 PMC8450055

[23] K. W. Nam , G. T. Oh , E. K. Seo , K. H. Kim , U. Koo , S. J. Lee , W. Mar , J. Ethnopharmacol. 2009, 123, 250. 19429369 10.1016/j.jep.2009.03.017

[24] P. Georg , R. Astaburuaga‐Garcia , L. Bonaguro , S. Brumhard , L. Michalick , L. J. Lippert , T. Kostevc , C. Gabel , M. Schneider , M. Streitz , V. Demichev , I. Gemund , M. Barone , P. Tober‐Lau , E. T. Helbig , D. Hillus , L. Petrov , J. Stein , H. P. Dey , D. Paclik , C. Iwert , M. Mulleder , S. K. Aulakh , S. Djudjaj , R. D. Bulow , H. E. Mei , A. R. Schulz , A. Thiel , S. Hippenstiel , A. E. Saliba , et al., Cell 2022, 185, 493. 35032429 10.1016/j.cell.2021.12.040 PMC8712270

[25] V. Hornung , F. Bauernfeind , A. Halle , E. O. Samstad , H. Kono , K. L. Rock , K. A. Fitzgerald , E. Latz , Nat. Immunol. 2008, 9, 847. 18604214 10.1038/ni.1631 PMC2834784

[26] H. Gong , Y. Chen , M. Chen , J. Li , H. Zhang , S. Yan , C. Lv , Front. Med. (Lausanne) 2022, 9, 1043859. 36452899 10.3389/fmed.2022.1043859 PMC9701739

[27] G. Bellani , J. G. Laffey , T. Pham , E. Fan , L. Brochard , A. Esteban , L. Gattinoni , F. van Haren , A. Larsson , D. F. Mcauley , M. Ranieri , G. Rubenfeld , B. T. Thompson , H. Wrigge , A. S. Slutsky , A. Pesenti , JAMA, J. Am. Med. Assoc. 2016, 315, 788.

[28] S. S. Mahalingam , S. Jayaraman , A. Arunkumar , H. M. Dudley , D. D. Anthony , C. L. Shive , J. M. Jacobson , P. Pandiyan , Front. Immunol. 2023, 14, 1231087. 37799713 10.3389/fimmu.2023.1231087 PMC10548880

[29] P. Pan , M. Shen , Z. Yu , W. Ge , K. Chen , M. Tian , F. Xiao , Z. Wang , J. Wang , Y. Jia , W. Wang , P. Wan , J. Zhang , W. Chen , Z. Lei , X. Chen , Z. Luo , Q. Zhang , M. Xu , G. Li , Y. Li , J. Wu , Nat. Commun. 2021, 12, 4664. 34341353 10.1038/s41467-021-25015-6 PMC8329225

[30] C. A. Dinarello , J. W. van der Meer , Semin. Immunol. 2013, 25, 469. 24275598 10.1016/j.smim.2013.10.008 PMC3953875

[31] H. J. Lachmann , I. Kone‐Paut , J. B. Kuemmerle‐Deschner , K. S. Leslie , E. Hachulla , P. Quartier , X. Gitton , A. Widmer , N. Patel , P. N. Hawkins , N. Engl. J. Med. 2009, 360, 2416. 19494217 10.1056/NEJMoa0810787

[32] R. C. Coll , A. A. Robertson , J. J. Chae , S. C. Higgins , R. Munoz‐Planillo , M. C. Inserra , I. Vetter , L. S. Dungan , B. G. Monks , A. Stutz , D. E. Croker , M. S. Butler , M. Haneklaus , C. E. Sutton , G. Nunez , E. Latz , D. L. Kastner , K. H. Mills , S. L. Masters , K. Schroder , M. A. Cooper , L. A. O'Neill , Nat. Med. 2015, 21, 248. 25686105 10.1038/nm.3806 PMC4392179

[33] C. Marchetti , B. Swartzwelter , F. Gamboni , C. P. Neff , K. Richter , T. Azam , S. Carta , I. Tengesdal , T. Nemkov , A. D'Alessandro , C. Henry , G. S. Jones , S. A. Goodrich , L. J. St , T. M. Jones , C. L. Scribner , R. B. Barrow , R. D. Altman , D. B. Skouras , M. Gattorno , V. Grau , S. Janciauskiene , A. Rubartelli , L. Joosten , C. A. Dinarello , Proc. Natl. Acad. Sci. U S A 2018, 115, E1530. 29378952 10.1073/pnas.1716095115 PMC5816172

[34] H. He , H. Jiang , Y. Chen , J. Ye , A. Wang , C. Wang , Q. Liu , G. Liang , X. Deng , W. Jiang , R. Zhou , Nat. Commun. 2018, 9, 2550. 29959312 10.1038/s41467-018-04947-6 PMC6026158

[35] H. Xu , J. Chen , P. Chen , W. Li , J. Shao , S. Hong , Y. Wang , L. Chen , W. Luo , G. Liang , Acta Pharm. Sin. B 2023, 13, 678. 36873170 10.1016/j.apsb.2022.09.014 PMC9978959

[36] A. Abderrazak , D. Couchie , D. F. Mahmood , R. Elhage , C. Vindis , M. Laffargue , V. Mateo , B. Buchele , M. R. Ayala , G. M. El , T. Syrovets , M. N. Slimane , B. Friguet , T. Fulop , T. Simmet , H. K. El , M. Rouis , Circulation 2015, 131, 1061. 25613820 10.1161/CIRCULATIONAHA.114.013730

[37] I. V. Hochheiser , M. Pilsl , G. Hagelueken , J. Moecking , M. Marleaux , R. Brinkschulte , E. Latz , C. Engel , M. Geyer , Nature 2022, 604, 184. 35114687 10.1038/s41586-022-04467-w

[38] V. Kluck , T. Jansen , M. Janssen , A. Comarniceanu , M. Efde , I. W. Tengesdal , K. Schraa , M. Cleophas , C. L. Scribner , D. B. Skouras , C. Marchetti , C. A. Dinarello , L. Joosten , Lancet Rheumatol. 2020, 2, 270. 10.1016/s2665-9913(20)30065-5 PMC7523621 33005902

[39] H. J. Whan , L. B. Gon , K. Y. Kee , Y. J. Woo , J. H. Kyoung , S. Hong , L. H. Young , L. K. Ro , L. H. Woo , Br. J. Pharmacol. 2001, 133, 503. 11399667 10.1038/sj.bjp.0704099 PMC1572810

[40] G. Lyss , T. J. Schmidt , I. Merfort , H. L. Pahl , Biol. Chem. 1997, 378, 951. 9348104 10.1515/bchm.1997.378.9.951

[41] W. L. Vande , M. Lamkanfi , Nat. Rev. Drug Discovery 2024, 23, 43. 38030687 10.1038/s41573-023-00822-2

[42] C. Chen , C. Wang , R. Pang , H. Liu , W. Yin , J. Chen , L. Tao , J. Transl. Med. 2023, 21, 278. 37098551 10.1186/s12967-023-04112-8 PMC10127506

[43] Y. Muto , E. E. Dixon , Y. Yoshimura , H. Wu , K. Omachi , N. Ledru , P. C. Wilson , A. J. King , O. N. Eric , M. G. Gunawan , J. J. Kuo , J. H. Cox , J. H. Miner , S. L. Seliger , O. M. Woodward , P. A. Welling , T. J. Watnick , B. D. Humphreys , Nat. Commun. 2022, 13, 6497. 36310237 10.1038/s41467-022-34255-z PMC9618568

[44] Y. Qin , G. Yan , Y. Qiao , D. Wang , C. Tang , BMC Genomics 2023, 24, 788. 38110868 10.1186/s12864-023-09892-3 PMC10729354

[45] G. Monaco , B. Lee , W. Xu , S. Mustafah , Y. Y. Hwang , C. Carre , N. Burdin , L. Visan , M. Ceccarelli , M. Poidinger , A. Zippelius , D. M. J. Pedro , A. Larbi , Cell Rep. 2019, 26, 1627. 30726743 10.1016/j.celrep.2019.01.041 PMC6367568

[46] H. Sharif , L. Wang , W. L. Wang , V. G. Magupalli , L. Andreeva , Q. Qiao , A. V. Hauenstein , Z. Wu , G. Nunez , Y. Mao , H. Wu , Nature 2019, 570, 338. 31189953 10.1038/s41586-019-1295-z PMC6774351

[47] C. Yu , J. Moecking , M. Geyer , S. L. Masters , J. Mol. Biol. 2018, 430, 142. 28733143 10.1016/j.jmb.2017.07.012

[48] J. Cui , W. Huang , B. Wu , J. Jin , L. Jing , W. P. Shi , Z. Y. Liu , L. Yuan , D. Luo , L. Li , Z. N. Chen , J. L. Jiang , J. Pathol. 2018, 245, 41. 29431199 10.1002/path.5054 PMC5947728

[49] Y. Li , N. Liu , Y. Qian , C. Jiao , J. Yang , X. Meng , Y. Sun , Q. Xu , W. Liu , J. Cui , W. Guo , J. Pharmacol. Sci. 2023, 152, 210. 37344056 10.1016/j.jphs.2023.05.004

[50] G. M. Ren , J. Li , X. C. Zhang , Y. Wang , Y. Xiao , X. Y. Zhang , X. Liu , W. Zhang , W. B. Ma , J. Zhang , Y. T. Li , S. S. Tao , T. Wang , K. Liu , H. Chen , Y. Q. Zhan , M. Yu , C. Y. Li , C. H. Ge , B. X. Tian , G. F. Dou , X. M. Yang , R. H. Yin , Sci. Immunol. 2021, 6, abe2933.

[51] S. Zhao , M. Yang , W. Zhou , B. Zhang , Z. Cheng , J. Huang , M. Zhang , Z. Wang , R. Wang , Z. Chen , J. Zhu , H. Li , Proc. Natl. Acad. Sci. U S A 2017, 114, 7245. 10.1073/pnas.1704155114 PMC5584424 28808021

[52] H. Xian , Y. Liu , N. A. Rundberg , R. Gatchalian , T. R. Crother , W. G. Tourtellotte , Y. Zhang , G. R. Aleman‐Muench , G. Lewis , W. Chen , S. Kang , M. Luevanos , D. Trudler , S. A. Lipton , P. Soroosh , J. Teijaro , J. C. de la Torre , M. Arditi , M. Karin , E. Sanchez‐Lopez , Immunity 2021, 54, 1463. 34115964 10.1016/j.immuni.2021.05.004 PMC8189765

[53] H. Tao , H. Zhao , A. Mo , L. Shao , D. Ge , J. Liu , W. Hu , K. Xu , Q. Ma , W. Wang , W. Wang , H. Cao , M. Mu , X. Tao , J. Wang , Ecotoxicol. Environ. Saf. 2023, 249, 114359. 36508797 10.1016/j.ecoenv.2022.114359

