# Supplementary material for: Inhibition of RACK1‐Mediated NLRP3 Oligomerization (Active Conformation) Ameliorates Acute Respiratory Distress Syndrome
Source: Adv Sci (Weinh). 2025 May 11;12(27):2411355. doi: 10.1002/advs.202411355 (PMC12279247; doi:10.1002/advs.202411355)
Supplement: Supplementary file 1 — Supporting Information [file ADVS-12-2411355-s002.docx]

**Supporting Information**


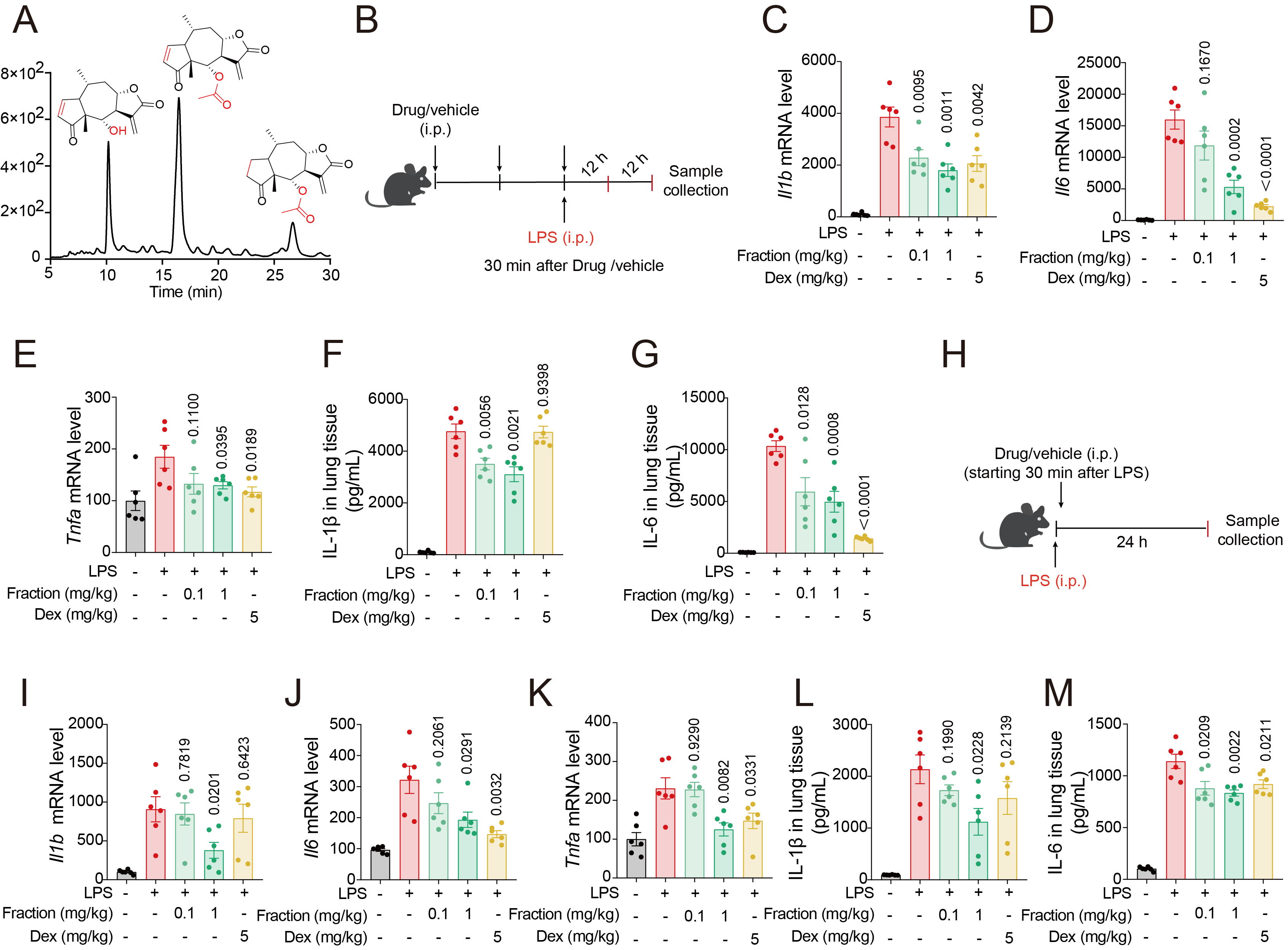


**Figure S1** The abundance of sesquiterpene lactone in *Inula helianthus aquatica* alleviates LPS-induced ARDS in mice. (A) The abundance of sesquiterpene lactone in *Inula helianthus aquatica*. (B) The scheme of prophylactic administration bigelovin or dexamethasone in ARDS induced by LPS. (C-E) qRT-PCR analysis of *Il1b* (C), *Il6* (D) and *Tnfa* (E) mRNA expression in lung tissues from above mice. (F and G) ELISA analysis of IL-1β (F) and IL-6 (G) levels of lung tissues from above mice. (H) The scheme of therapeutic administration of bigelovin or dexamethasone in ARDS induced by LPS. (I-K) qRT-PCR analysis of *Il1b* (I), *Il6* (J) and *Tnfa* (K) mRNA expression in lung tissues from above mice. (L and M) ELISA analysis of IL-1β (L) and IL-6 (M) levels of lung tissues from above mice. Data were presented as mean ± SEM and were representative of six independent experiments. Statistical significance was assessed by two-tailed unpaired *t* test.


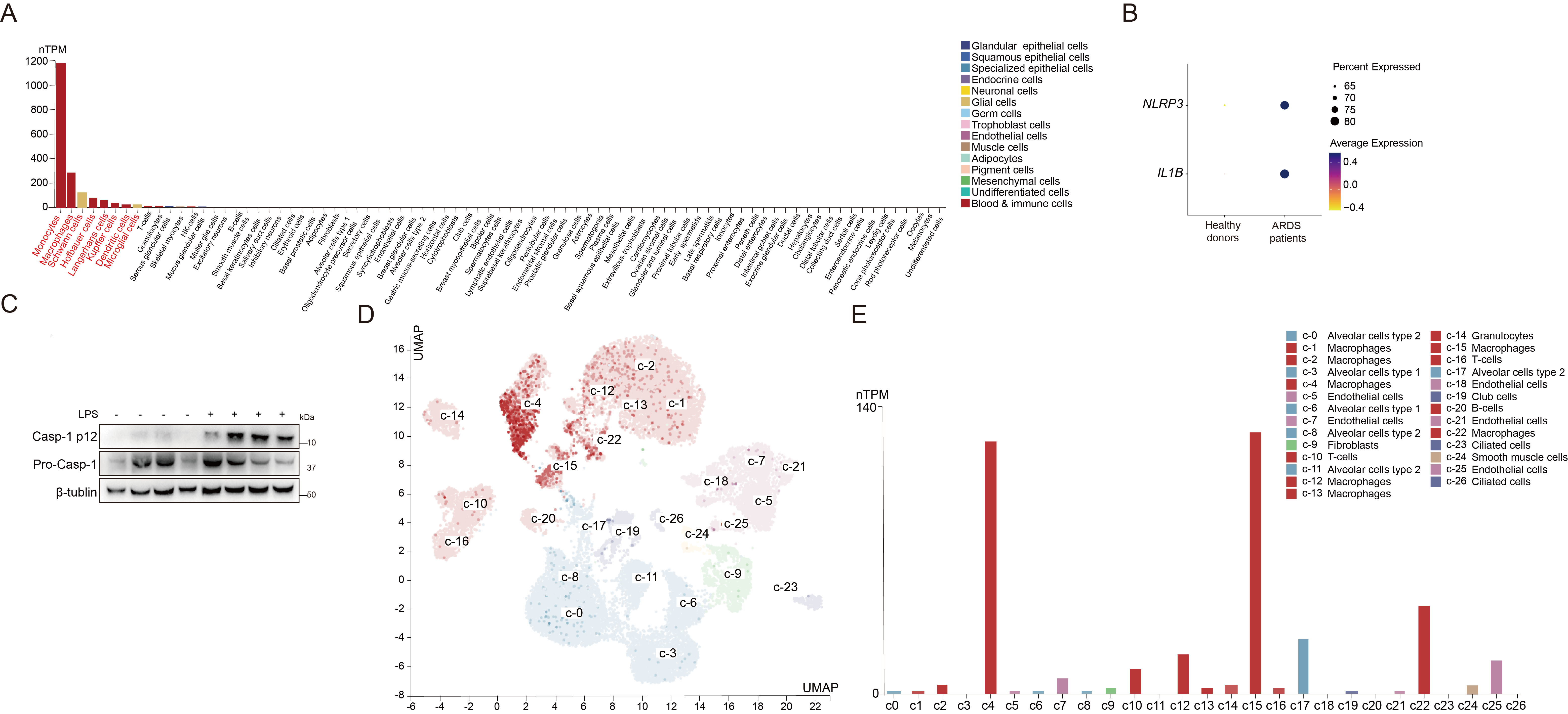


**Figure S2** Gene expression omnibus analysis of NLRP3. (A) Single cell type expression cluster of *Nlrp3*. (B) Bubble plots showing the expression of NLRP3 and IL-1B in single-cell data (GSE175450). The darker the color and the larger the circle are, the higher the expression. (C) Western blotting analysis of caspase-1 (p12) of mice induced by ARDS. (D and E) The UMAP plot (D) and color scale (E) of cell type of *Nlrp3* in lung tissues.


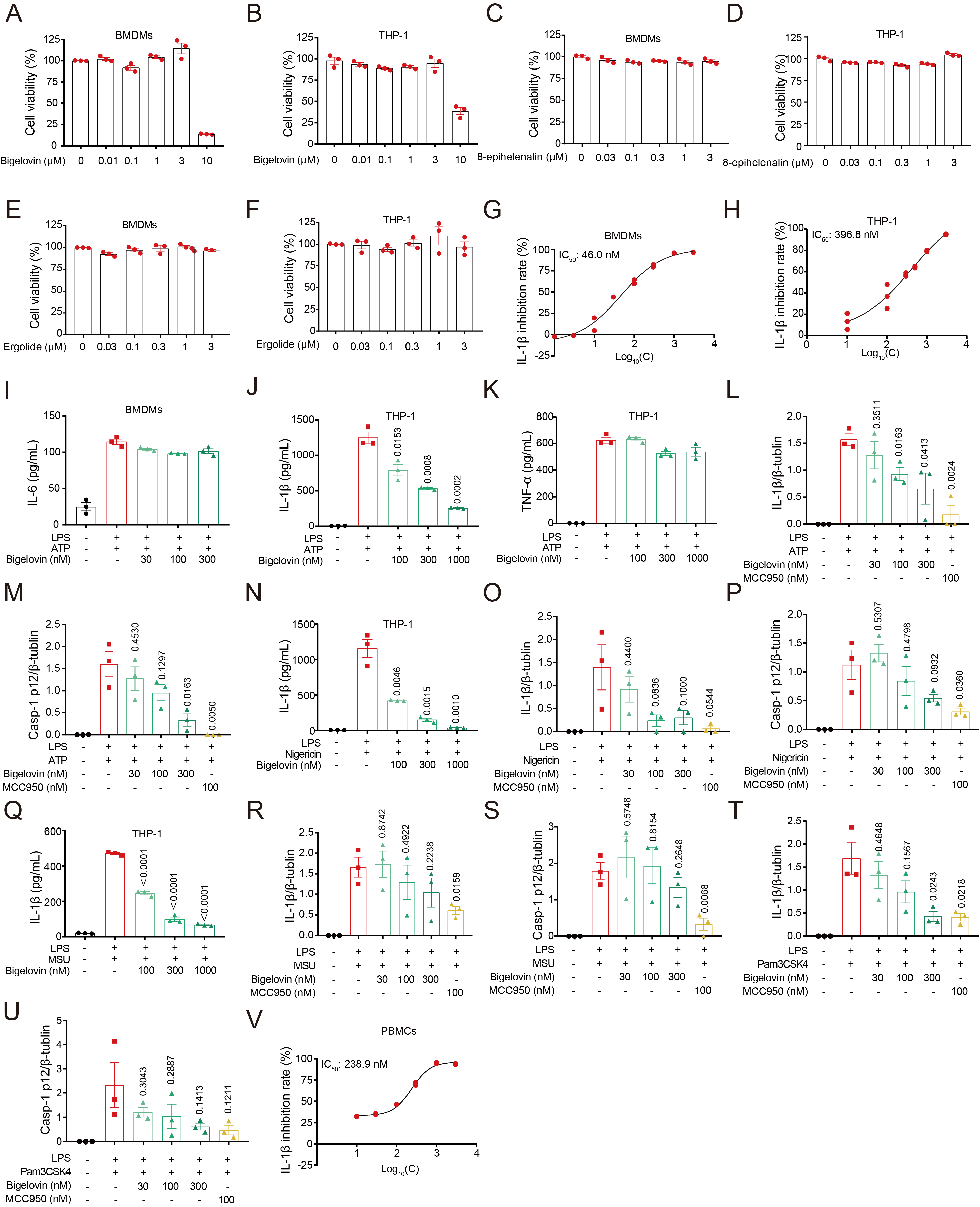


**Figure S3** Bigelovin inhibits NLRP3 inflammasome activation in BMDMs, THP-1, and PBMCs. (A-F) The cell viability of BMDMs or THP-1 cells treatment with bigelovin (A and B), 8-epihelenalin (C and D), and Ergolide (E and F) at different concentrations for 24 h. (G and H) ELISA analysis of IL-1β in culture supernatants of LPS-primed BMDMs (G) or THP-1 (H) treated with bigelovin before stimulation with ATP. (I) ELISA analysis of IL-6 in culture supernatants of LPS-primed BMDMs treated with bigelovin before stimulation with ATP. (J and K) ELISA analysis of IL-1β (J) or TNF-α (K) in culture supernatants of LPS-primed THP-1 treated with bigelovin before stimulation with ATP. (L and M) Quantitative analysis of cleaved IL-1β (L) or caspase-1 (p12) (M) described in Figure 2D. (N) ELISA analysis of IL-1β in culture supernatants of LPS-primed THP-1 treatment with bigelovin before stimulation with nigericin. (O and P) Quantitative analysis of cleaved IL-1β (O) and caspase-1 (p12) (P) described in Figure 2G. (Q) ELISA analysis of IL-1β in culture supernatants of LPS-primed THP-1 treated with bigelovin before stimulation with MSU. (R and S) Quantitative analysis of cleaved IL-1β (R) or caspase-1 (p12) (S) described in Figure 2I. (T and U) Quantitative analysis of IL-1β (T) or caspase-1 (p12) (U) described in Figure 2K. (V) ELISA analysis of IL-1β in culture supernatants of PBMCs treated with bigelovin and then stimulated with LPS for 16 h. Data were presented as mean ± SEM and were representative of three independent experiments. Statistical significance was assessed by two-tailed unpaired *t* test.


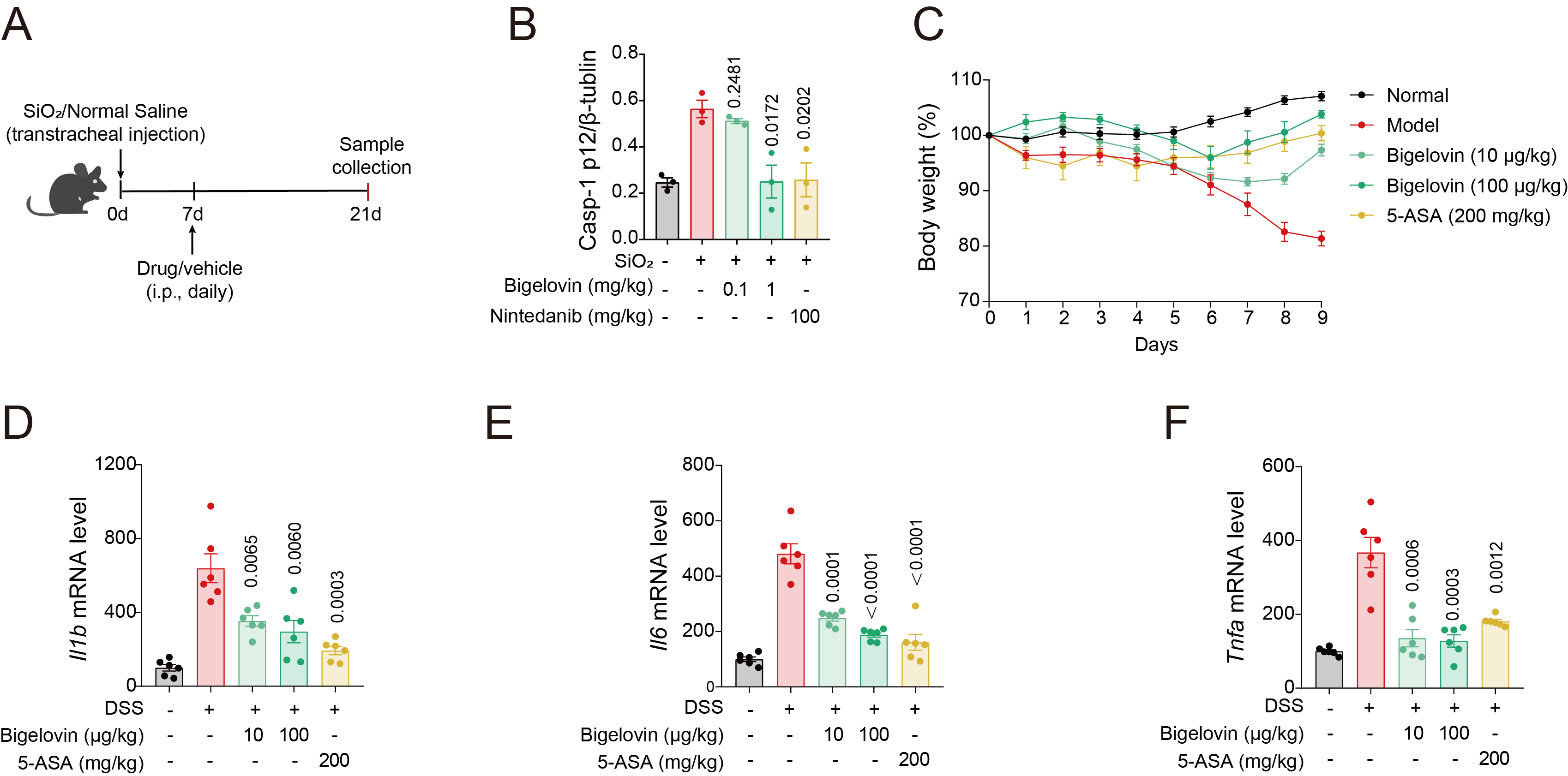


**Figure S4** Bigelovin inhibits NLRP3 inflammasome activation *in vivo*. (A) Schematic procedure of bigelovin treatment on silicosis mice. (B) Quantitative analysis of the immublot of Casp-1 p12 described in Figure 3G (n = 3). (C) Body weight loss (n = 6). (D-F) Gene expression of inflammatory factors (*Il1b*, *Il6*, *Tnfa*) in colon levels were assessed by qRT-PCR (n = 6). Data were presented as mean ± SEM statistical significance was assessed by two-tailed unpaired *t* test.


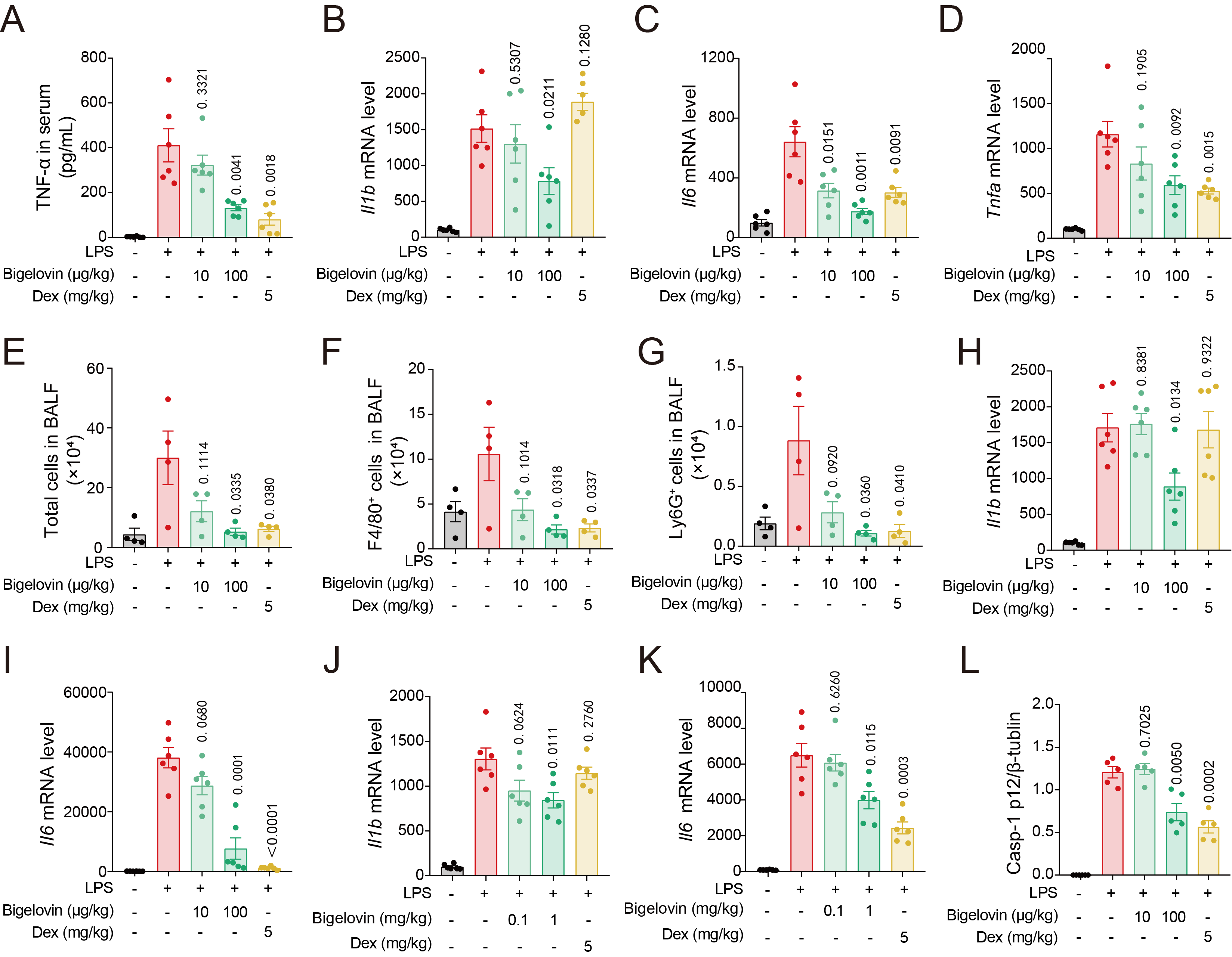


**Figure S5** Bigelovin alleviates LPS-induced ARDS in mice. (A-D) Mice were prophylactic intraperitoneal injection of bigelovin and then challenged with 7.5 mg/kg LPS for 12 h (n = 6). (A) ELISA analysis of TNF-α levels of serum from above mice w. (B-D) qRT-PCR analysis of *Il1b* (B), *Il6* (C) and *Tnfa* (D) mRNA expression in lung tissues from above mice. (E-G) Mice were prophylactic intraperitoneal injection of bigelovin and then challenged with 7.5 mg/kg LPS for 12 h (n = 4). (E) Total cells, (F) macrophages, (G) neutrophils, were quantified in BALF fluid. (H and I) Mice were treated with 7.5 mg/kg LPS and then injected intraperitoneally bigelovin for 24 h (n = 6). qRT-PCR analysis of *Il1b* (H) and *Il6* (I) mRNA expression in lung tissues from above mice. (J and K) Mice were prophylactic intragastric administration of bigelovin and then challenged with 7.5 mg/kg LPS for 12 h (n = 6). qRT-PCR analysis of *Il1b* (J) and *Il6* (K) mRNA expression in lung tissues from above mice. (L) Quantitative analysis of the immunoblot of caspase-1 (p12) described in Figure 4D (n = 5). Data were presented as mean ± SEM and statistical significance was assessed by two-tailed unpaired *t* test.


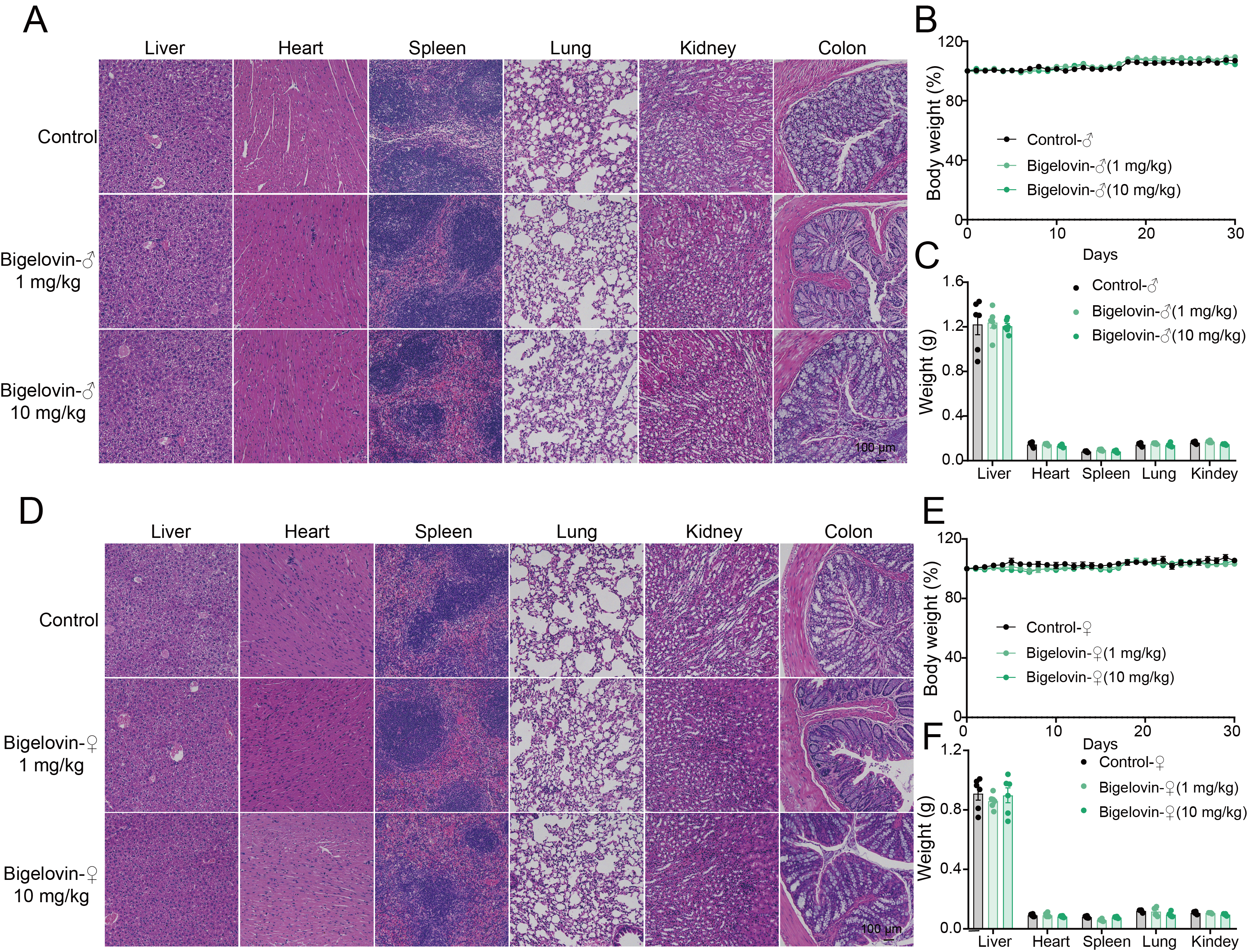


**Figure S6** Biosafety evaluation of bigelovin. (A-C) Male C57BL/6J mice were intragastric administration of bigelovin (1 mg/kg, 10 mg/kg) continuously for 30 days. (A) H&E staining of liver, heart, spleen, lung, kidney, and colon tissues from above mice (scale bar = 100 μm). (B) Body weight analysis of mice treated with bigelovin (1 mg/kg, 10 mg/kg). (C) The weight of liver, heart, spleen, lung, and kidney tissues from above mice. (D-F) Female C57BL/6J mice were intragastric administration of bigelovin continuously (1 mg/kg, 10 mg/kg) for 30 days. (D) H&E staining of liver, heart, spleen, lung, kidney, and colon tissues from above mice (scale bar = 100 μm). (E) Body weight analysis of mice treated with bigelovin (1 mg/kg, 10 mg/kg). (F) The weight of liver, heart, spleen, lung, and kidney tissues from above mice. Data were presented as mean ± SEM and were representative of six independent experiments.


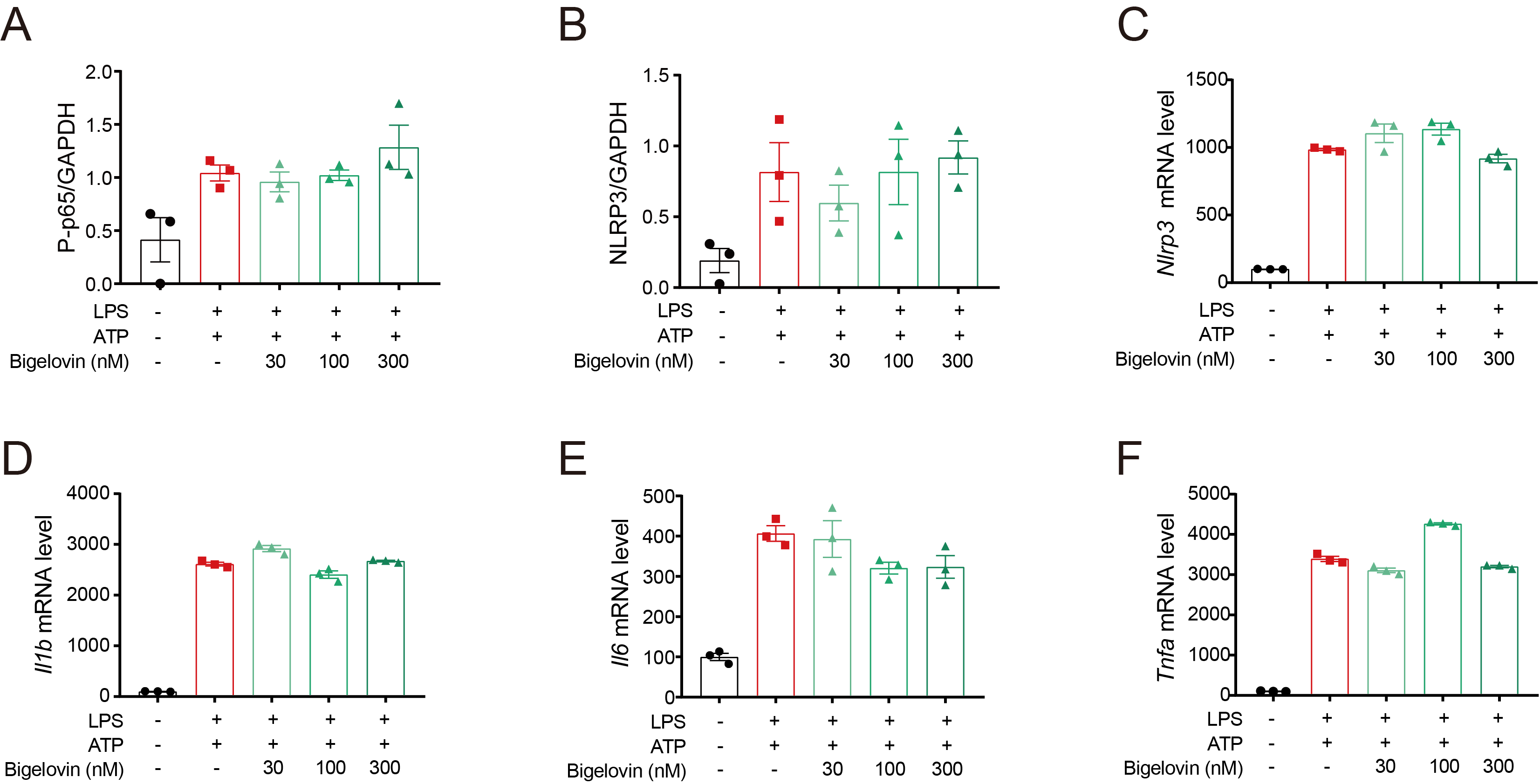


**Figure S7** Function of bigelovin in LPS-induced priming. (A and B) Quantitative analysis of the immunoblot of p-p65/GAPDH (A) or NLRP3/GAPDH (B) described in Figure 5C. (C-F) *Nlrp3* (C), *Il1b* (D), *Il6* (E), and *Tnfa* (F) mRNA expression in LPS-primed BMDMs treated with bigelovin before stimulation with ATP. Data were presented as mean ± SEM and were representative of three independent experiments. Statistical significance was assessed by two-tailed unpaired *t* test.


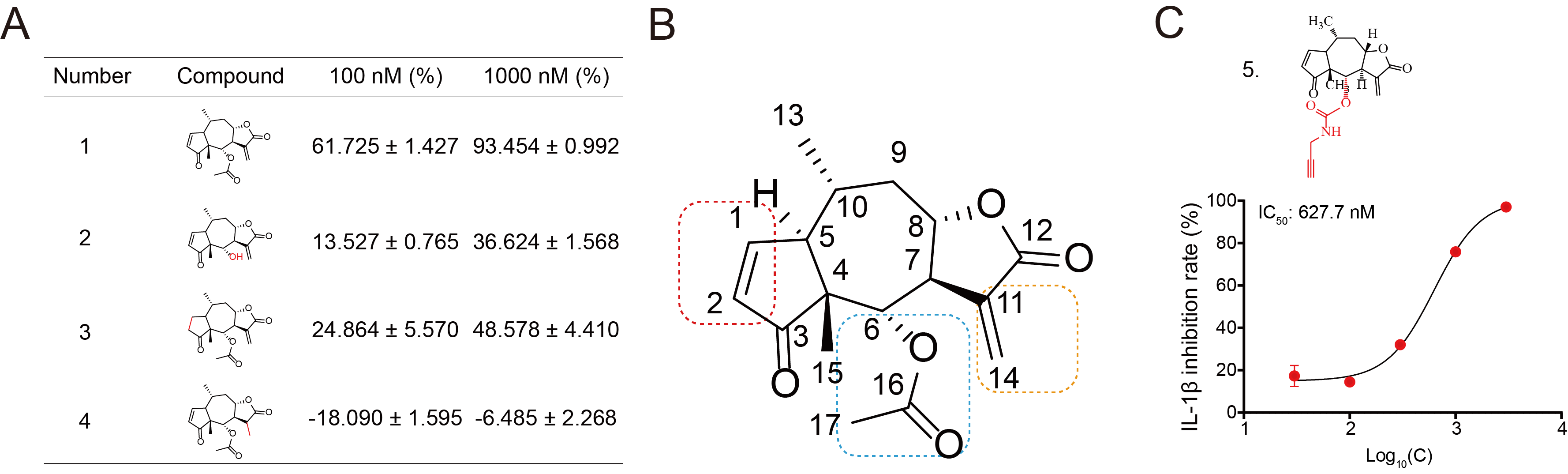


**Figure S8** Structure activity relationship of bigelovin derivatives. (A) The inhibitory rate of IL-1β in culture supernatants of LPS-primed BMDMs treated with compound before stimulation with ATP were measured by ELISA (n = 3). (B) Structure activity relationship of bigelovin. (C) ELISA analysis of IL-1β in culture supernatants of LPS-primed BMDMs treated with the biotin labeled bigelovin before stimulation with ATP (n = 3). Data were presented as mean ± SEM.

**
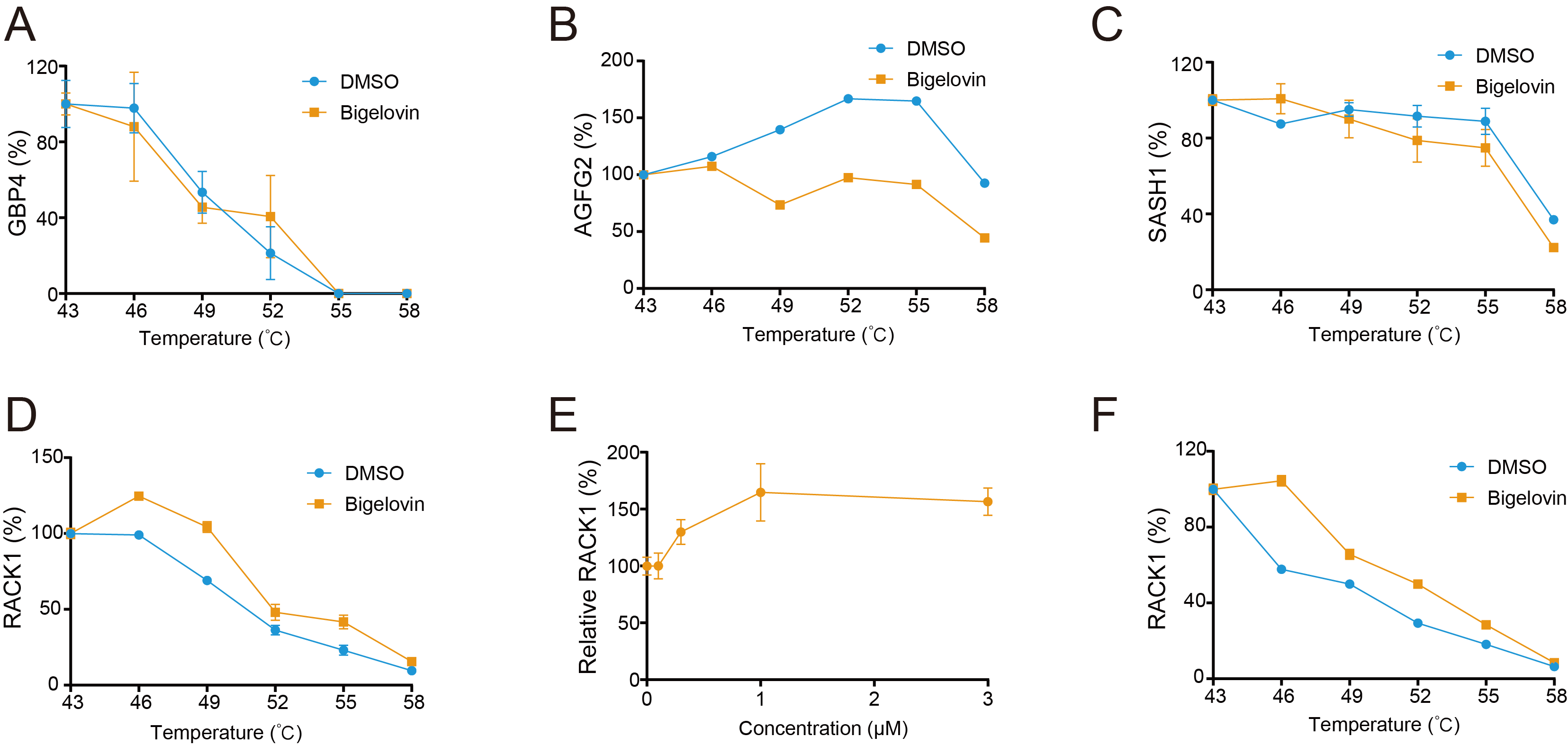
**

**Figure S9** Bigelovin binds to RACK1 protein. (A-C) Quantitative analysis of the immublot of Gbp4 (A), Agfg2 (B) and Sash1 (C) described in Figure 7C and 7D. (D-F) Quantitative analysis of RACK1 described in Figure 7E, 7F, and 7I. Data were presented as mean ± SEM and were representative of three independents.


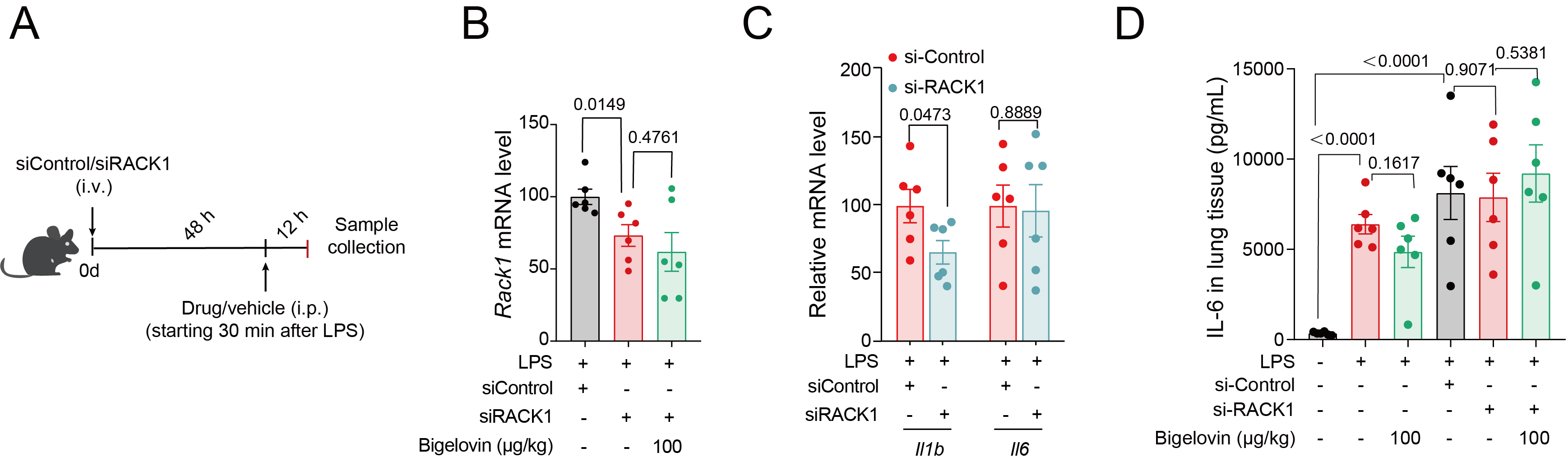


**Figure** **S10** Bigelovin inhibits NLRP3 inflammasome mainly by binding to RACK1. (A) The scheme of LPS-induced ARDS mice knocked down RACK1. (B and C) qRT-PCR analysis of *Rack1* (B) and *Il1b, Il6* (C) in lung tissues from above mice transfected with control siRNA or siRNA against *Rack1*. (D) ELISA analysis of IL-6 levels of lung tissues from above mice. Data were presented as mean ± SEM and were representative of six independent experiments. Statistical significance was assessed by two-tailed unpaired *t* test.

**Figure S11.** **Spectroscopic data of chemical**

**8-epi-helenalin**: C_15_H_18_O_4_, white powder; ^1^H NMR (500 MHz, CD_3_OD) *δ* 7.79 (dd, *J* = 5.9 and 2.0 Hz 1H), 6.26 (d, *J* = 3.6 Hz, 1H), 6.16 (dd, *J* = 6.0 , 2.8 Hz, 1H), 6.10 - 5.99 (m. 1H), 4.55 (ddd, *J* = 11.8 , 10.2 ,d 3.0 Hz, 1H), 4.17 (dd, *J* = 8.3 , 2.2 Hz, 1H), 3.01 (dt, *J* = 10.8 , 2.4 Hz, 1H), 2.93 (ddt, *J* = 10.2 , 8.2 , 3.4 Hz, 1H) 2.54 (ddd, *J* = 13.1 , 4.6 , 2.9 Hz, 1H), 2.26 - 2.00 (m, 2H), 1.57 - 1.47 (m, 1H), 1.29 (d, *J* = 6.6 Hz, 3H), 1.20 (s, 3H)。ESI-MS (m/z): [M+H]^+^ = 263.1;

^1^H NMR spectrum (500 MHz) of 8-epi-helenalin in CDCl_3_

**Bigelovin**: C_17_H_20_O_5_, white powder; ^1^H NMR (500 MHz, CDCl_3_) δ 7.70 (d, *J* = 7.9 Hz, 1H), 6.22 (s, 1H), 6.11 (d, *J* = 8.9 Hz, 1H), 5.91 (d, *J* = 3.2 Hz, 1H), 5.61 (d, *J* = 7.5 Hz, 1H), 4.61 (d, *J* = 25.1 Hz, 1H), 3.11 – 2.99 (m, 2H), 2.57 (d, *J* = 20.5 Hz, 1H), 2.06 (d, *J* = 34.2 Hz, 1H), 1.96 (s, 3H), 1.54 (dt, *J* = 13.2, 11.9 Hz, 1H), 1.28 (d, *J* = 6.6 Hz, 3H), 1.21 (s, 3H), ESI-MS (m/z): [M+H]^+^ = 305.1;

^1^H NMR spectrum (500 MHz) of Bigelovin in CDCl_3_

**Ergolide**: C_17_H_22_O_5_, white powder; ^1^H NMR (500 MHz, CDCl_3_) δ 6.23 (d, *J* = 3.5 Hz, 1H), 5.87 (d, *J* = 3.2 Hz, 1H), 5.52 (d, *J* = 7.8 Hz, 1H), 4.51 (d, *J* = 24.8 Hz, 1H), 3.06 (d, *J* = 24.6 Hz, 1H), 2.53 (d, *J* = 20.4 Hz, 1H), 2.45 (s, 1H), 2.31 (d, *J* = 28.5 Hz, 1H), 2.17 (d, *J* = 54.4 Hz, 2H), 2.00 (s, 3H), 1.85 (s, 1H), 1.50 (d, *J* = 60.5 Hz, 2H), 1.14 (d, *J* = 6.6 Hz, 3H), 1.09 (s, 3H), ESI-MS (m/z): [M+H]^+^ = 307.1。

^1^H NMR spectrum (500 MHz) of Ergolide in CDCl3

**Table S1.** **Patient Characteristics with Direct ARDS**

|  | Mild ARDS  (n = 28) | Moderate ARDS  (n = 35) | Sereve ARDS  (n = 5) | *P*  Value |
| --- | --- | --- | --- | --- |
| Age, yr | 75.7 ± 1.6 | 68.7 ± 1.9 | 68.4 ± 7.7 | 0.0356 |
| Sex, M, % | 75.0 | 71.4 | 80.0 |  |
| Leukocyte, x10^9/L | 10.9 ± 0.8 | 11.5 ± 1.1 | 11.2 ± 2.4 | 0.9253 |
| Lactate, mmol/L | 1.5 ± 0.1 | 1.7 ± 0.2 | 1.1 ± 0.2 | 0.3927 |
| SOFA score | 8.5 ± 0.9 | 8.0 ± 1.6 | 10 | 0.8604 |
| Apache ll score | 25.2 ± 0.9 | 26.4 ± 3.5 | 19 | 0.5553 |
| Death in lCU, % | 17.9 | 20.0 | 40.0 |  |
| PaO2/FiO2 ratio | 239.4 ± 5.6 | 158.6 ± 5.1 | 92.4 ± 4.1 | <0.0001 |
| PEEP | 6.5 ± 0.3 | 7.6 ± 0.4 | 7.0 ± 0.7 | 0.0771 |
| Tidal Volume | 475.0 ± 11.3 | 458.8 ± 13.1 | 457.5 ± 42.5 | 0.6475 |

Definition of abbreviations: APACHE II = Acute Physiology and Chronic Health Evaluation II. ARDS = acute respiratory distress syndrome; PEEP = positive endexpiratory pressure; SOFA = sepsis-related organ failure assessment. Data are presented as mean ± SEM. Statistical significance was assessed by one-way ANOVA.
